# Global Genetic Response in a Cancer Cell: Self-Organized Coherent Expression Dynamics

**DOI:** 10.1371/journal.pone.0097411

**Published:** 2014-05-15

**Authors:** Masa Tsuchiya, Midori Hashimoto, Yoshiko Takenaka, Ikuko N. Motoike, Kenichi Yoshikawa

**Affiliations:** 1 Institute for Advanced Biosciences, Keio University, Tsuruoka, Japan; 2 Systems Biology Program, School of Media and Governance, Keio University, Fujisawa, Japan; 3 Graduate School of Frontier Science, The University of Tokyo, Kashiwa, Japan; 4 Nanosystem Research Institute, National Institute of Advanced Industrial Science and Technology, Tsukuba, Japan; 5 Tohoku Medical Megabank Organization, Tohoku University, Sendai, Japan; 6 Faculty of Life and Medical Sciences, Doshisha University, Kyotanabe, Japan; National Scientific and Technical Research Council (CONICET), Argentina

## Abstract

Understanding the basic mechanism of the spatio-temporal self-control of genome-wide gene expression engaged with the complex epigenetic molecular assembly is one of major challenges in current biological science. In this study, the genome-wide dynamical profile of gene expression was analyzed for MCF-7 breast cancer cells induced by two distinct ErbB receptor ligands: epidermal growth factor (EGF) and heregulin (HRG), which drive cell proliferation and differentiation, respectively. We focused our attention to elucidate how global genetic responses emerge and to decipher what is an underlying principle for dynamic self-control of genome-wide gene expression. The whole mRNA expression was classified into about a hundred groups according to the root mean square fluctuation (*rmsf*). These expression groups showed characteristic time-dependent correlations, indicating the existence of collective behaviors on the ensemble of genes with respect to mRNA expression and also to temporal changes in expression. All-or-none responses were observed for HRG and EGF (biphasic statistics) at around 10–20 min. The emergence of time-dependent collective behaviors of expression occurred through bifurcation of a coherent expression state (CES). In the ensemble of mRNA expression, the self-organized CESs reveals distinct characteristic expression domains for biphasic statistics, which exhibits notably the presence of criticality in the expression profile as a route for genomic transition. In time-dependent changes in the expression domains, the dynamics of CES reveals that the temporal development of the characteristic domains is characterized as autonomous bistable switch, which exhibits dynamic criticality (the temporal development of criticality) in the genome-wide coherent expression dynamics. It is expected that elucidation of the biophysical origin for such critical behavior sheds light on the underlying mechanism of the control of whole genome.

## Introduction

One of the fundamental challenges in life science is to unveil the basic mechanism that underlies how the genome regulates the activity of tens of thousands of genes in an autonomous manner. The recent remarkable success with iPS cells by the ectopic expression of key transcription factors [1] has opened the door not only to possible manipulation of the cell fate to any cell state through somatic genome reprogramming, but also to understanding the genetic mechanism of development and disease in vitro. The eukaryotic genome (epigenome) defines a cell state by determining dynamically which genes are activated or arrested; the cellular process organizes the exceedingly complex molecular structure on giant DNA molecules with the cooperation of nuclear proteins accompanied by dynamic epigenetic modifications [2]. However, our present understanding of fundamental questions, such as how can the genome, which is a highly complex molecular system, self-regulate genome-wide genetic activity and what is the guiding principle by which the genome drives cell differentiation and reprogramming, is still in its infancy.

With regard to the control of gene regulation, it has been reported that several hundreds or thousands of genes in a yeast cell culture are up-regulated within a few minutes in a rapid and genome-wide transcriptional response [3], [4]; in embryonic mammalian stem cells, a few key transcription factors (Oct4, Sox2, and Nanog) coordinate the expression of thousands of genes with epigenetic modifications accompanied by epigenetic molecules such as chromatin regulators [5].

When we scrutinize the dynamics of biochemical reactions associated with gene expression, the expression of each gene in a population of cells (e.g., MCF-7 human cancer cells in this report) carries stochastic noise stemming from intra- (intrinsic) and inter-cellular (extrinsic) processes [6]–[12]. This stochasticity has constructive (correlated) and destructive effects (uncorrelated) on biological processes including the regulation of gene expression. There exist two underlying difficulties to understand the robust on/off control of gene expression in a cell: noise in stochastic expression and noise due to the heterogeneity of cell types (cell-to-cell variability) in the population.

The presence of stochastic noise (due to the intrinsic effect of a low copy number of mRNAs of a gene per cell) suggests bringing about instability in abundance of genetic product if the system is based solely on a very large number of specific key-lock interactions (i.e., if we do not include the molecular environment) [13]. In addition to the noisy low copy number effect, there may not be enough statistical number of molecules within the small space of the cell nucleus: the breakdown of the central limit theorem upon binding equilibrium between key and lock molecules [14] can increase the stochastic (destructive) effect due to random collision between reactants (e.g., RNA polymerase and DNA) on the dynamics of gene expression. These intrinsic effects should cause the breakdown of the collective ensemble behavior of gene expression.

The extrinsic effect of cell-to-cell interaction might generate non-genetic heterogeneity (cell-to-cell variability generated by the same set of genes) [15], [16]. Different processes in different cell types in the population might cause further instability in the robust control of the expression of a large number of genes in the cell. In contrast, another report suggested that cell-to-cell variability is largely the result of deterministic regulatory processes, rather than stochasticity at the single-cell level [17]. A recent theoretical study showed that extrinsic noise plays an important role in the heterogeneity within a population of cells through phenotype switching in the regulation of gene expression [12]. Further research will be needed to elucidate the essential cause of the heterogeneity in a cell population.

Until recently, the most widely accepted mechanism for the self-regulation of gene expression has been a genetic network composed of a large number of specific interactions, i.e., key-lock complex molecular interactions; gene expression is a dynamic process of unwrapping DNA from histone molecules and exposing a specific region of double-stranded DNA to produce mRNA from DNA sequence information for a certain gene to carry out a specific cellular process. The staggeringly complex cellular system that arises from the dynamic key-lock molecular interactions with intrinsic and extrinsic stochastic environments raises a fundamental question, despite the underlying stochastic nature and heterogeneous environment: How can a cell with a small, compact nucleus provide robust control of coordinated genome-wide gene expression [13]?

**Figure 1:**
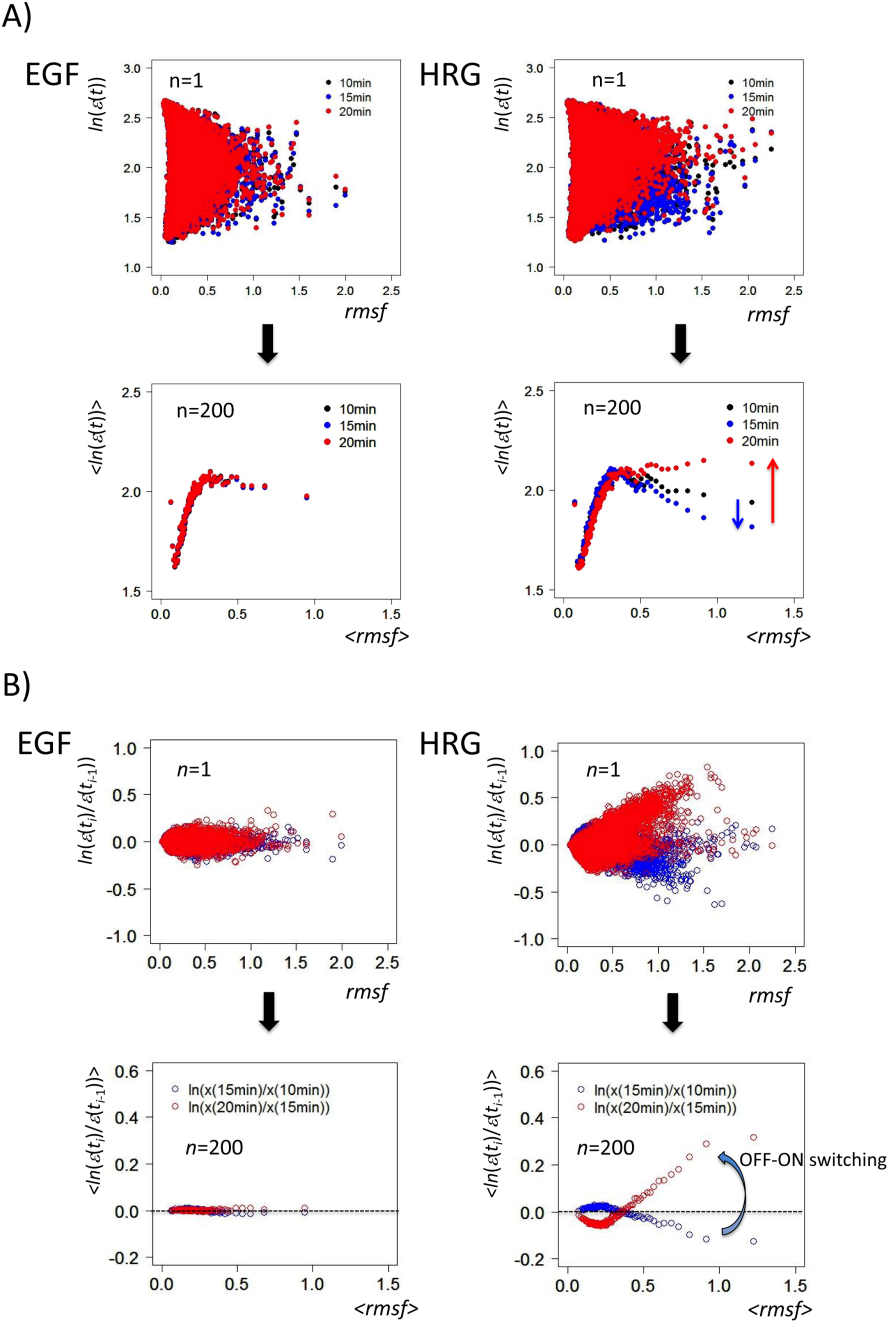
Emergence of biphasic *dynamic emergent averaging behaviors* (DEABs) of the expression and the expression change. The transition from scattered expression (first row; *N* = 22035) to time-dependent correlation (second row) is shown as the collective behavior of ensemble groups: DEAB of A) the expression (symbolically represented by *ln*(*ε*(*t*)); called simply ‘the expression’) and B) the expression change (*ln*(*ε*(*t_i_*)/*ε*(*t_i_*_–1_))). The image shows biphasic genomic responses (biphasic statistics) to HRG and EGF; plots of single mRNA (*n* = 1; first row) and a group of genes (*n* = 200; second row) for A) the expression at *t* = 10 min (black dot), 15 min (blue), and 20 min (red), and B) the expression change from *t_i_*_–1_ = 10 min to *t_i_* = 15 min (blue cycle) and from *t_i_*_–1_ = 15 min to *t_i_* = 20 min (red), reflects OFF-ON switching down- to up-regulation). Brackets around *x,* <*x*>, reflect the simple arithmetic mean of *x* in a group (*n* = 200).

To tackle this fundamental question, we may wish to pay attention to the ‘noise’ of gene expression in genome-wide data through the quantitative evaluation of stochastic fluctuation to shed light on the hidden mechanism of genome-wide global self-regulation. We investigated the whole transcriptome activity considering the entire set of genes, i.e., the expression of all genes obtained from microarray data, and sorted all mRNA expression (usually on the order of tens of thousands) according to the degree of the change in temporal expression from a baseline, and formed groups of mRNA expression (see Methods). Through this grouping of gene expression in different biological processes, we have uncovered emergent nonlinear correlations (e.g., Figure 1) [18]–[20]: i.e., global nonlinear correlations between ensemble (group) averages of temporal changes in expression and mRNA expression with an increase in *n* (see details in File S1). These global correlations indicate the presence of averaging (mean field) behavior in large and stochastic genetic activity as hidden genome-scale collective behavior. It is well known that the existence of mean field behavior suggests the presence of a governing principle in physical many-body (e.g., molecular) systems; thus, genetic mean field behaviors suggest the existence of underlying principles that are ‘sensed’ by the genome as a whole with the consequent emergence of collective modes that encompass the coordinated activity of thousands of genes [Tsuchiya M, Hashimoto M, Tomita M, Yoshikawa K, Giuliani A, “Collective Genome-Wide Expression Modes: Major Roles of Low-Variance Genes”, unpublished].

The emerging picture of gene-expression regulation can be explained in terms of a highly integrated dynamical system in a multidimensional phase space spanned by the expression levels of the whole set of genes. Notably, even mRNA species with very low signal intensities allow for the global reconstruction of cell population dynamics, as in the case of hematopoietic stem cell differentiation [21], which is consistent with the picture arising from an analysis of the entire transcriptome, which strongly suggests the presence of a set of constraints that allow the genome to act as a highly coherent/cooperative system (‘genome field’ [22]). A similar profile that resembles the emergent nonlinear correlation accompanied by fluctuation can occur for the distribution of single-gene expression for cells in culture when the intrinsic (uncorrelated) noise becomes low [6].

In this report, we analyzed the whole genome expression dynamics (22035 probes and 18 time points; Methods) that accompanied MCF-7 (human breast cancer) cell proliferation and differentiation through the activation of ErbB receptor by epidermal growth factor (EGF) and heregulin (HRG), respectively. HRG induces cell differentiation by sustaining extracellular signal-regulated kinase (ERK) activity to produce a significant phosphorylated transcription factor, c-Fos activation, while EGF stimulates cell proliferation by inducing transient ERK activity with negligible c-Fos induction [23]. A biphasic signaling response in respect to ErB receptor signaling dynamics with differences at the c-Fos level was elucidated [23]–[25].

To understand whether there are distinct genomic responses in relation to the biphasic signaling response, we conducted a comprehensive analysis of the whole genomic response for both HRG and EGF ligand activation on the ErbB receptor of MCF-7 breast cancer cells by grouping genes based on time-dependent changes in expression. To elucidate how global genetic responses emerge and further to decipher what is an underlying principle for dynamic self-control of genome-wide gene expression, we focused on time-dependent global genetic responses for the first 30 min after ligand activation, which shows the biphasic genomic responses (biphasic statistics) to EGF and HRG.

In the following sections, we demonstrate that the essential scenario on self-organized expression dynamics through bifurcation of ensemble of coherent expression reveals how genome-wide expression is coordinated differently (all-or-none) in cell proliferation (EGF) and differentiation (HRG). Most importantly, we address the presence of criticality as a route for genomic transition and its dynamical change (dynamic criticality) in collective behaviors of mRNA expression, which give us a thought-provoking insight to understand how a cell in population can conduct the robust dynamic control of genome-wide coordinated gene expression for a short time, even within the small, packed nuclear space. Finally, we discuss a potential biophysical origin of criticality from the conformal transition of genomic DNA that controls transcriptional activity through a structural transition [26], [27].

## Results

### Global Genetic Response Led by Group Dynamics of Genes: Dynamic Emergent Averaging Behaviors (DEABs)

We investigated whole transcriptome activity in MCF-7 cancer cells stimulated by HRG-beta and EGF at 18 time points (*t* = 0, 10, 15, 20, 30, 45, 60, 90[min], 2, 3, 4, 6, 8, 12, 24, 36, 48, *t_T_* = 72[h]), and considered the expression of all probes (*N* = 22035; Gene Expression Omnibus database ID: GSE13009; see Methods) assigned to each gene or open reading frame (ORF) in microarray data; we call such probes ‘mRNA expression’, which includes the expression of genes as well as the expression of variants of mRNA. Two ligands, EGF and HRG, activate ErbB family receptors to produce distinct cell fates (cell differentiation and cell proliferation, respectively) by provoking different signal durations which leads to the ligand-specific biphasic production of c-Fos proteins after 20 min. EGF provokes transient ERK activation, while HRG induces sustained ERK activation, causing all-or-none (i.e., all for HRG and none for EGF) responses of the phosphorylated transcription factor c-Fos [23], [25].

To clarify whether these two ligands can induce distinct genomic activities by better understanding the transcriptome expression dynamics, we grouped mRNA expression according to the standard deviation of time-dependent fluctuation in expression (*root mean square fluctuation*: *rmsf*) for all of the probes without any filtering of the original data (see Methods). As the group size (*n* = 1, 100, 200, 300) increased, a nonlinear correlation emerged from scattered points at time *t*, where the fluctuation of groups from these asymptotic correlations reduced as the grouping size *n* increased [19]. Dynamic emergent averaging (collective) behaviors (DEABs) were noticed in the profile of *rmsf* against logarithm of mRNA expression, <*rmsf*> versus <*ln*(*ε*(*t_i_*))> (Figure 1A) or *rmsf* against temporal change in logarithm of mRNA expression, <*rmsf*> versus <*ln*(*ε*(*t_i_*)/*ε*(*t_i_*_−1_))> (Figure 1B), where the brackets, < >, denote the ensemble/group average, and *ε*(*t_i_*) reflects mRNA expression at time *t_i_* (*i = *0,1,.,17). Hereafter, we refer to the logarithm of mRNA expression and the temporal change in logarithm of mRNA expression as *the expression* and *the expression change*, respectively.

*DEAB of the expression* at time *t* (Figure 1A) revealed a nonlinear correlation between groups based on average values of expression and *rmsf* at a fixed time point. When we compare DEAB of the expression between different time points, coordinated motion of the ensemble of mRNA expression emerges according to the degree (i.e., standard deviation) of temporal fluctuation of mRNA expression (i.e., *rmsf*). In Figure 1A, DEABs of the expression at three time points (10, 15, and 20 min) show a clear difference between EGF and HRG: for EGF induction, they overlap, with no apparent change, while for HRG induction, in some groups (<*rmsf*> >0.42) distinct slopes were seen at three time points, and an especially sharp change from a negative to a positive slope was seen between 15 min and 20min, while there were no changes in the other groups (<*rmsf*> <0.42). Thus, in DEAB of the expression, there appear to be dynamic and static ensembles of mRNA expression; a more rigorous definition of expression ensembles is given in the following section.

On the other hand, *DEAB of the expression change* shows a marked change with time between different time points (Figure 1B), indicating which groups are up- or down-regulated in a coordinated manner. As shown in Figure 1B, an all-or-none response is also seen in the regulation of mRNA expression; in the EGF response, DEABs are nearly balanced (i.e., nearly zero average change in expression), whereas in the HRG response, for <*rmsf*> >0.42, the corresponding DEAB shows a marked change from down-regulation (10–15 min) to up-regulation (15–20 min). In contrast, DEAB for <*rmsf*> <0.42 changed from nearly balanced or up-regulation to down-regulation. Thus, in the HRG response, the dynamic changes in DEABs of both the expression (Figure 1A) and the expression change (Figure 1B) were consistent for <*rmsf*> >0.42, while changes in opposite directions were seen for <*rmsf*> <0.42. These temporal changes in DEAB of the expression and the expression change will be addressed as autonomous coherent expression dynamics.

Next, we investigated the frequency distributions of mRNA expression according to DEAB to understand the biophysical phenomena that underlie gene expression dynamics. Profiles that include thousands of mRNAs can provide information on the biophysical laws that underlie mRNA expression dynamics, such as the Gaussian distribution for Brownian dynamics and power-law behavior for scale-free interaction. Furthermore, the changes in profiles, e.g., a change from unimodal to bimodal, might reveal some critical phenomena [28]–[30].

**Figure 2:**
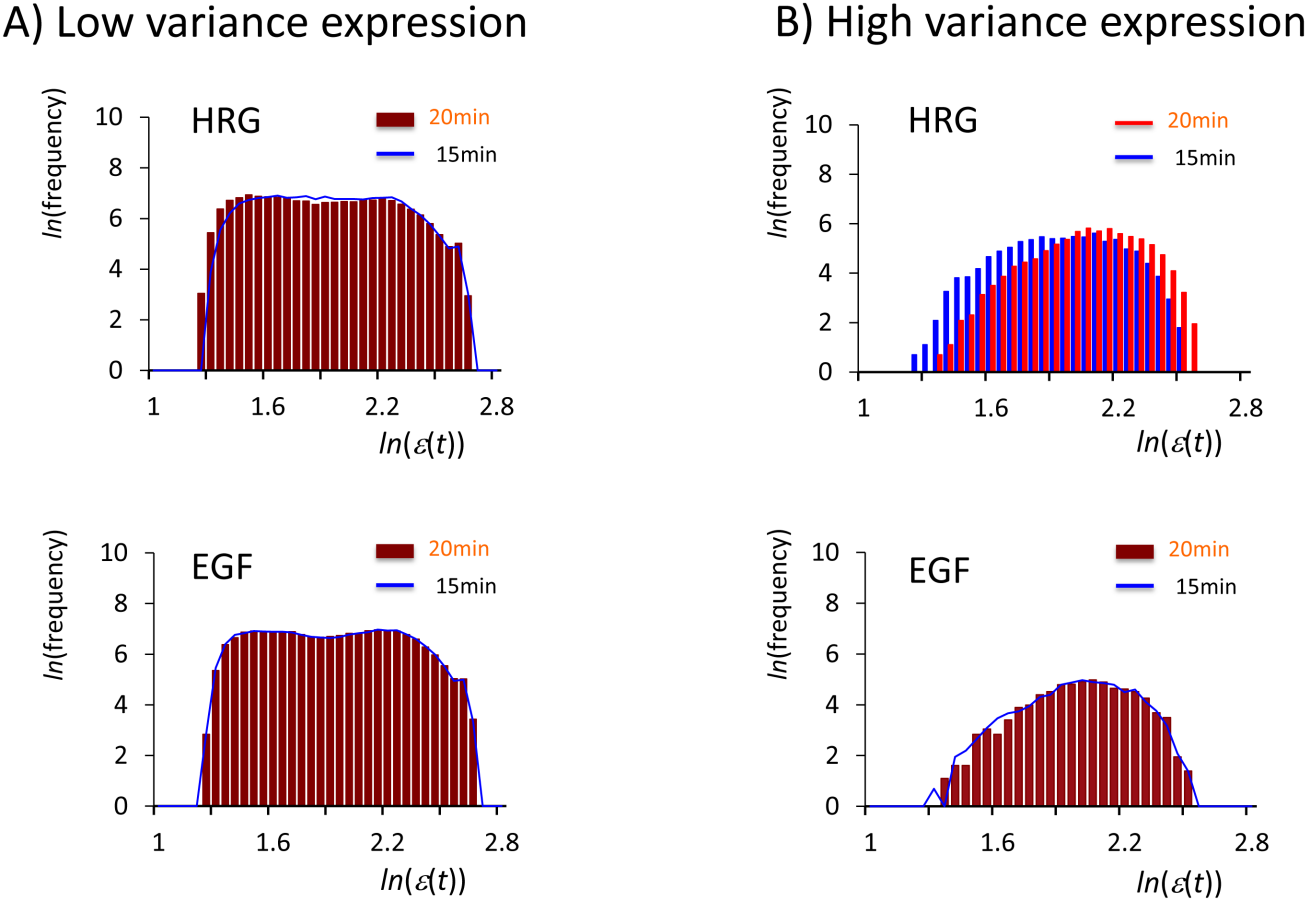
Unimodal to bimodal frequency distribution for DEAB of the expression. The profiles of the frequency distribution of the expression (*ln*(*ε*(*t*))) from 15 min to 20 min change from unimodal to bimodal for A) high-variance expression (the root mean square fluctuation, *rmsf* >0.42) and B) low-variance expression (*rmsf* <0.42). First row: the HRG response for *rmsf* >0.42 shows a peak-shift of unimodal profiles from *t = *15 min (blue histogram) to *t* = 20 min (red) with a change in the lower to higher value of the expression, while binomial frequency distributions between 15 min (blue polygonal line) and 20 min (red histogram) almost perfectly overlap each other for *rmsf* <0.42. Second row: the EGF response shows almost the perfect overlap of profiles for both unimodal (*rmsf* >0.42) and bimodal (*rmsf* <0.42) distributions, which suggests that there is no temporal averaging response, consistent with DEAB of the expression for the EGF response (Figure 1A). For all histograms in this report, the bin size is set to 0.05.

In Figure 2, the frequency histograms of expression (15–20 min) change from a unimodal (*rmsf* >0.42) to bimodal distribution (*rmsf* <0.42) for both the EGF and HRG ensemble groups. Interestingly, in the HRG response, the frequency distributions for *rmsf* >0.42 between 15 min and 20 min do not overlap; the profile is deformed with a shift in the peak from *ln*(*ε*(15 min))  = 1.8 to *ln*(*ε*(20 min))  = 2, which is called the *unimodal shift*. Otherwise, the frequency distributions at 15 min and 20 min almost overlap each other.

DEABs showed genome-wide dynamic correlations for both the expression and the expression change. The results of the entire transcriptome analysis suggest the presence of a set of constraints that allow the genome to act as a coherent/coordinated system. We will now consider the biophysical significance of the dynamic motion of DEABs of the expression that is accompanied by a change from a unimodal to a bimodal frequency distribution.

### Bifurcation of Coherent Expression States in DEAB of the Expression: Characteristic Expression Domains Revealed

To understand how a global response emerges and then to elucidate its underlying principle, we need to understand how gene expression is self-organized on a genome-wide scale. We used a density analysis to visualize the dynamics of up- or down-regulation between different time points. A density analysis of the clustering of noisy gene-expression profiles has been shown to be robust [31]. We applied a Gaussian kernel as a density analysis on the space spanned by the expression versus the change in expression (‘regulatory space’). Since the most dramatic response was observed between 15 min and 20 min, in this section we focus on analyzing the dynamics of mRNA expression in DEABs of the expression from 15 to 20 min.

**Figure 3:**
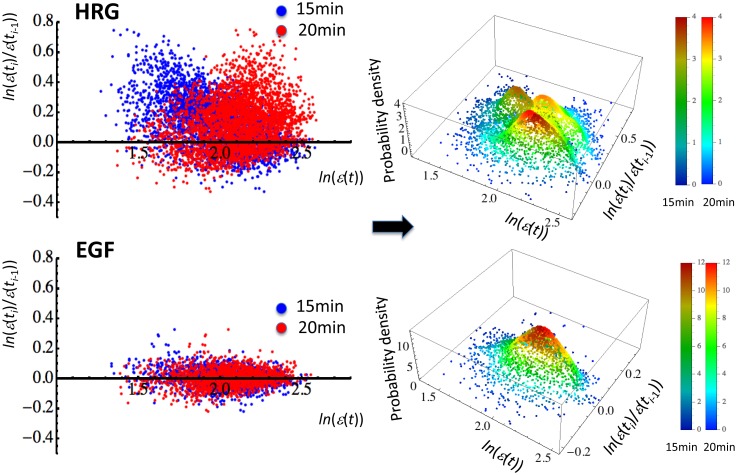
Existence of coherent expression states (CESs) as hill-like functions. Plots of single mRNA expression for *rmsf* >0.42 (blue dot: 15 min and red dot: 20 min) are superimposed in the left panel (first row: 3269 expressions for HRG; second row: 1482 for EGF). In the right panel, the probability density function (PDF) using a Gaussian kernel by Mathematica 9 (default setting) for each point (left panel) reveals hill-like functions in pseudo-3-dimensional space (genetic landscape; z-axis: probability density). Superimposition of the genetic landscapes between *t_i–_*_1_ = 15 min and *t_i_* = 20 min - first row: the HRG response has three CESs; two independent CESs plus one CES that results from the overlap of CESs between 15 min (darker color) and 20 min (lighter color) around a zero change in expression at the y-axis; second row: the EGF response has a single CES as the overlap of two CESs around a zero change in expression. The legend shows a lighter (darker) color bar at 20 min (at 15 min) for PDF.

Given an expression value at time *t* (*t* = 15 min or 20 min), the regulatory space shows whether mRNA expression at time *t* is up-regulated, down-regulated, or balanced during this period. Interestingly, if we evaluate the probability density function (PDF) for the regulatory space and take the probability density on the z-axis, the pseudo-3-dimensional-plot shows hill-like functions to reveal the density landscape (“genetic landscape”) of the expression dynamics (Figure 3). In the HRG response, there are two hill-like functions at each time point; if we superimpose the genetic landscapes between 15 min and 20 min, we see three independent hill-like functions for *rmsf* >0.42 (*n* = 3269 mRNAs): two distinct hill-like functions for each time point and one that results from the overlapping of two temporally (almost) invariant hill-like functions. In contrast, in the EGF response (*rmsf* >0.42; *n* = 1482), a single hill-like function does not show an apparent temporal change. Thus, up- or down-regulated mRNA expressions form up- or down-regulated hills on the landscape and their dynamic changes reflect the coherent expression behavior of thousands of mRNAs; a hill-like function on the genetic landscape is considered to be a coherent expression state (CES). To further confirm the existence of CES, we analyzed the dynamic motion of CESs (the next section).

**Figure 4:**
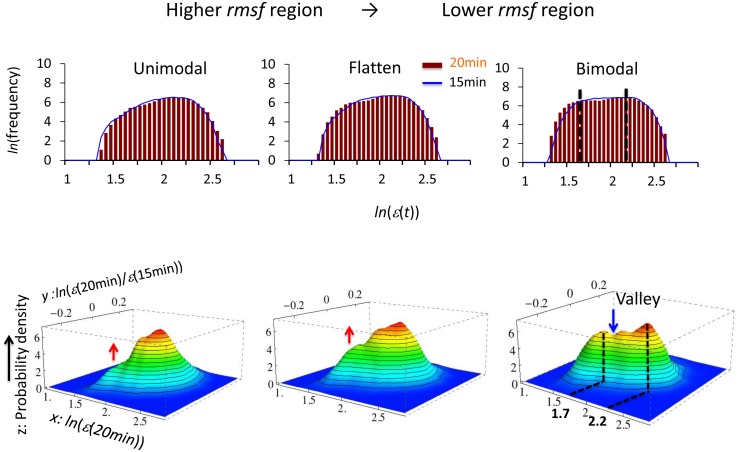
Onset of the bifurcation of CES for DEAB of the expression (HRG). The onset of bifurcation of a new CES as the growth of a hill-like function is shown. In the first row, the profile of the frequency distribution of expression changes from unimodal (0.26< *rmsf* <0.66; left) to bimodal (0.17< *rmsf* <0.57; right) through a flattened unimodal profile (0.22< *rmsf* <0.62; center). The genetic landscape (second row) for 15–20 min for each region of *rmsf* illustrates that the onset of bifurcation of CES transforms from a unimodal to bimodal profile; the red arrow (second row) points to the formation of CES and the blue arrow points to the formation of a valley, which gives rise to a low-expression state (LES). The peaks of the bimodal frequency distribution coincide with the highest density of CESs at around *ln*(*ε*)* = *1.7 and 2.2 (black dash lines).

**Figure 5:**
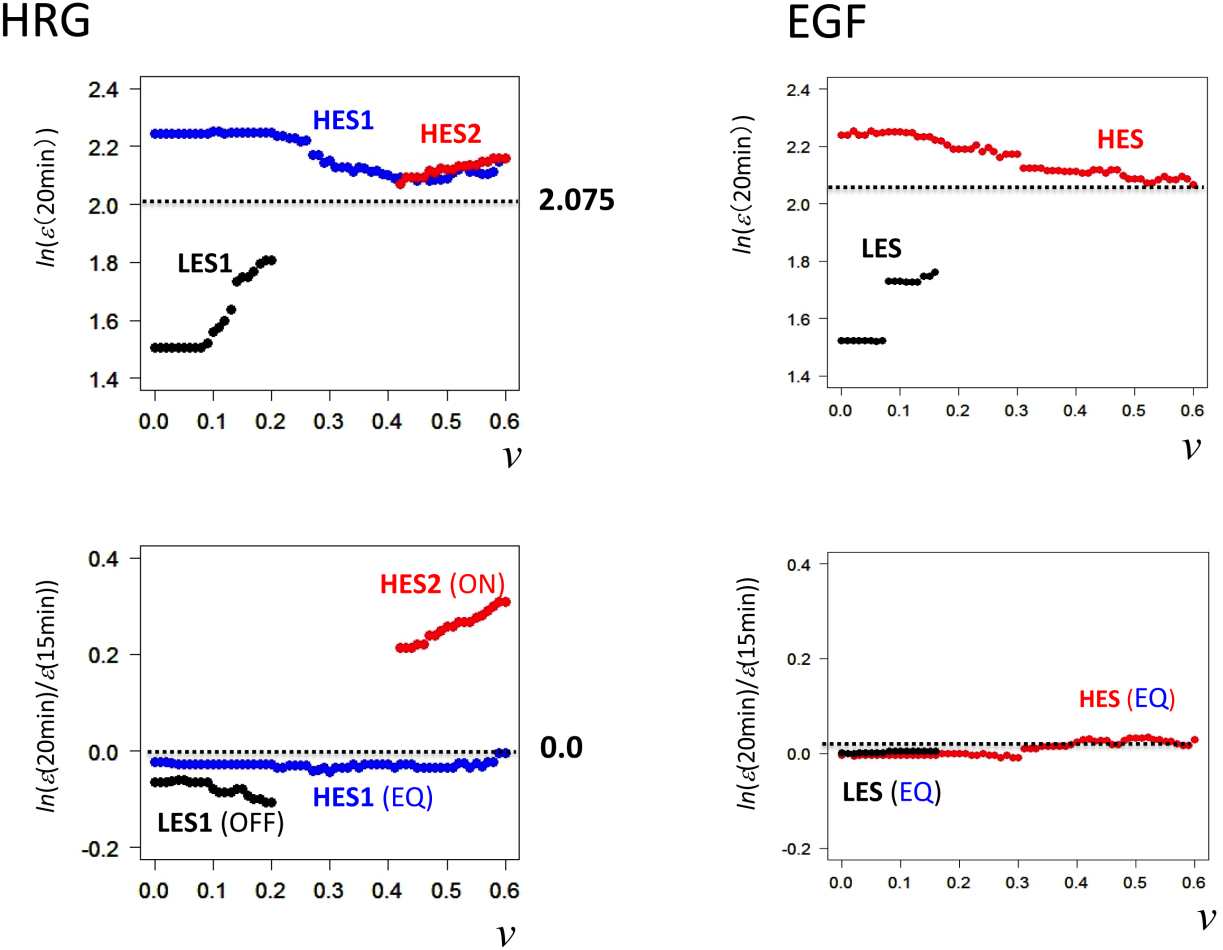
Bifurcation of CESs in DEAB of the expression. The bifurcations of CESs in DEAB of the expression for 15–20 min are examined with an incremental change in a segment, *v* < *rmsf* < *v* + *r,* as an extension of Figure 4, where the range *r* is set to 0.4 and *v* is a variable of *rmsf*. The bifurcation diagrams of the expression (*v* against the expression; first row) at *t* = 20 min, and the expression change (*v* against the change in the expression for 15–20 min; second row) are plotted for HRG (left panel) and EGF (right). The bifurcation diagram of the expression defines the expression level at *ln*(*ε*)  = 2.075 (lower: low- and upper: high-expression) because of the existence of a valley, which separates the low and high CESs (Figure 6), whereas the bifurcation diagram of the expression change shows three activity levels of CES: ON (positive change in the expression), EQ (near zero) and OFF (negative change in the expression). The bifurcation diagrams clearly show distinct characteristic expression domains between HRG and EGF: static, transit and dynamic domains for *rmsf* <0.21, 0.21< *rmsf* <0.42, and 0.42< *rmsf* for HRG, and static and transit domains for *rmsf* <0.16 and 0.16< *rmsf* for EGF (see details in the main text).

We investigated the formation of a CES in DEAB of the expression in terms of the incremental change in a segment with a certain range of *rmsf* (*v* < *rmsf* < *v* + *r*), where the range *r* is set to 0.4 so that it includes the expression of thousands of mRNAs, and *v* is a variable of *rmsf*. In Figure 4, the three segments of *rmsf* for the HRG response describe the onset of bifurcation of CES during the period 15–20 min, where a new CES bifurcated at the segment around 0.21< *rmsf* <0.61. A similar onset of bifurcation of CES at the segment around 0.16< *rmsf* <0.56 was observed in the EGF response (data not shown). Bifurcation diagrams of CESs as a function of *v* against mRNA expression (*bifurcation diagram in the expression*) or the change in the expression (*bifurcation diagram in the expression change*) were obtained by tracing the positions of hilltops of CESs (Figure 5). CESs are functions of the expression level and expression activity. Note that the position of a hilltop can depend on the choice of the kernel density, but the bifurcation property does not change in DEAB.

**Figure 6:**
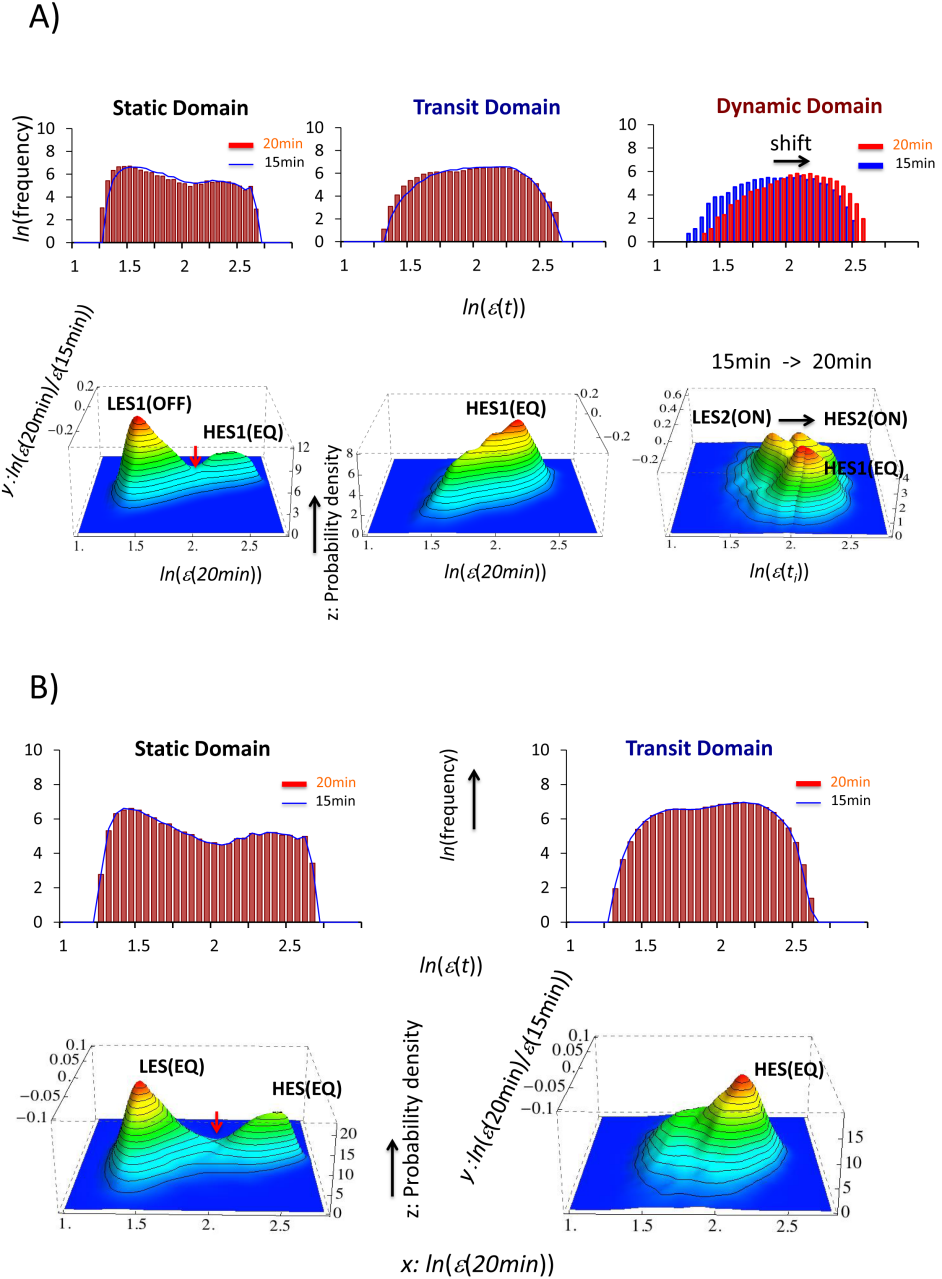
Genetic landscape of the characteristic expression domains. Each row (A: HRG and B: EGF) corresponds to frequency distributions of mRNA expression (first) and genetic landscapes (second: side view). In the genetic landscape, a static domain with a valley is characterized by two CESs: A) HES1(EQ) and LES1(OFF) for *rmsf* <0.21; and B) HES(EQ) and LES(EQ) for *rmsf* <0.16, a transit domain is characterized by A) HES1(EQ) for 0.21< *rmsf* <0.42; and B) HES(EQ) for *rmsf* >0.16, and a dynamic domain is characterized by three CESs: A) LES2(ON), HES2(ON) and HES1(EQ) for *rmsf* >0.42, which is the result of superimposition of the genetic landscapes between 15 min and 20 min (right panel in the second row); a state shift occurs from LES2(ON) at 15 min to HES2(ON) at 20 min, consistent with the unimodal shift of the frequency distribution (A: first row). The red arrow points to the valley to separate the low and high CESs. The activity level of a coherent expression state (ON, EQ and OFF) refers to Figure 5.

With regard to the expression level, the existence of a valley in the landscape between two hills of CES separates the expression levels into high and low at *ln*(*ε*) = 2.075 for both HRG and EGF (Figures 5 and 6). Moreover, a bifurcation in the regulatory space revealed that CES possesses three expression activities between 15 min and 20 min: up-regulated, down-regulated and equilibrated. The up- and down-regulations can be considered to be the ON and OFF activity levels in comparison to equilibrated (EQ) regulation, where the rates of mRNA production and decomposition are nearly balanced. Interestingly, in the EGF genomic response, all of the CESs are at the EQ level (Figure 5).

Most importantly, the bifurcation diagrams (Figure 5) during the period 15–20 min clearly show differences between the HRG and EGF genomic responses; the three characteristic domains in the HRG response can be categorized as i) *static domain* (*n* = 9059): *rmsf* <0.21 with two high-expression states (HES1(EQ) and HES2(ON), ii) *transit domain* (*n* = 9707): 0.21< *rmsf* <0.42 with a high-expression state (HES1(EQ)), and iii) *dynamic domain* (*n* = 3269): *rmsf* <0.42 with high- and low-expression states (HES1(EQ) and LES1(OFF)). In contrast, the EG response does not show a dynamic domain and only two domains are present: i) *static domain* (*n* = 7091): *rmsf* <0.16 with HES(EQ) and LES(EQ), and ii) *transit domain* (*n* = 14944): 0.16< *rmsf* with HES(EQ).

Regarding the naming of domains, Figure 1A for the HRG response showed ensemble of relatively small fluctuations with *rmsf* <0.42 and dynamic ensemble of relatively large fluctuations with *rmsf* >0.42. We named the ensemble with *rmsf* >0.42 as the dynamic domain. Whereas, the bifurcation analysis of CES (Figure 5 for HRG) indicates that the ensemble of small fluctuations (*rmsf* <0.42) is further categorized into two ensembles: one (0.21< *rmsf* <0.42) for the onset of CES with the flattened unimodal frequency profile as shown in Figure 4 and another (*rmsf* <0.21) for two CESs with the bimodal profile (Figure 6A); the former is called the transit domain and the latter, the static domain. The situation is essentially the same in the EGF response, except for the lack of dynamic domain.

A repeated experiment confirmed the characteristic domains during the period 15–20 min (File S2), and although different ensembles of mRNAs are being considered, the same naming of CES in the different domains can be applied when the continuity of CES holds in the bifurcation diagram in terms of the parameter *rmsf*. It would be interesting to determine whether a common genomic property is necessary for the continuity of CES upon bifurcation.

The emergence of a low-expression state in the static domains for both HRG and EGF corresponds to the formation of a bimodal frequency distribution of the expression (Figures 6A–B). It is important to realize that the bifurcation of CES (i.e., LES) occurred with a change from a unimodal to a bimodal density profile to generate low-expression state. Moreover, in the static domain, phase separation was observed between LES and HES through the valley, which suggests that the two separate states should be related to each other through a specific genomic DNA structure. Furthermore, as demonstrated in the following section, the dynamics of a pair of CESs (LES and HES) forms autonomous bistable switch (ABS): a pendulum oscillatory system. The only difference in the static domains between the HRG and EGF responses was in the activity level of the low-expression states, LES_HRG_(OFF) and LES_EGF_(EQ), which will be addressed in terms of the dynamic motion of LES in the next section.

Figure 6A shows that through a unimodal shift of the HRG frequency distribution, one of the two CESs at 15 min in the dynamic domain changed from a low- to a high-expression state (LES2 to HES2) at 20 min, while another high-expression state at 15 min, HES1(EQ), remained unchanged. The change from LES2 to HES2 explains the temporal ensemble motion of DEAB of the expression, with a change in the slope from negative to positive (Figure 1A).

Therefore, the biphasic statistics on DEAB of the expression (Figure 1A) between HRG and EGF can be explained by a) the bifurcation of LES2(ON) in the HRG dynamic domain through unimodal to bimodal profile of frequency distribution of mRNA expression, and b) the state change of CES from LES2(ON) to HES2(ON) around HES1(EQ), whereas in the EGF genomic response, all of the CESs remain in an equilibrated state during the period 15–20 min (Figure 6B). Table 1 summarizes the self-organized coherent expression through the bifurcation of CESs in DEAB of the expression, with differences in the characteristic domains between HRG and EGF.

**Table 1:** Bifurcations of CES, Characteristic Domains and Criticality at 15–20 min.

	HRG			EGF	
Criticality Domain	Above Dynamic	Near Transit	Below Static	Near Transit	Below Static
Range of *rmsf*	>0.42	0.21–0.42	<0.21	>0.16	<0.16
# of mRNAs	3269	9707	9059	14944	7091
Distribution:mRNA expression	Unimodal	FlattenedUnimodal	Bimodal	FlattenedUnimodal	Bimodal
DEAB of the expression	Up	No change	No change	No change	No change
Coherent Expression State: CES	HES1 (EQ)andHES2 (ON)	HES1 (EQ)	HES1 (EQ)andLES1 (OFF)	HES (EQ)	HES (EQ)andLES (OFF)
Bifurcation of CES	LES2 (ON)	Onset of birth of LES1	LES1 (OFF)	Onset of birth of LES	LES (OFF)
State change	LES2 (ON; 15 min)→HES2 (ON; 20 min)	-	-	**-**	-

### Dynamic Motion of CES in DEAB of the Expression Change: Autonomous Bistable Switch Revealed

In this section, we elucidate the dynamic relation between the motion of CES within a characteristic domain (Figures 6A–B) and DEABs of the expression change (Figure 1B). Importantly, the sorting of mRNA expression was based on the time-independent variable *rmsf*, such that the mRNA’s IDs (probe IDs) for a given characteristic domain do not change over time, but the expression of mRNAs is up- or down-regulated in a coherent manner. Especially, the phase separation of LES and HES in the static domains was confirmed, such that about 98% of mRNA expression within a specific CES (LES or HES) during the period 10–20 min was common between two different time points, thus ensuring the existence of CES. Notably, the dynamics of gene expression within a CES is stochastic; i.e., the coherent behavior emerges only from the ensemble of mRNA expression.

**Figure 7:**
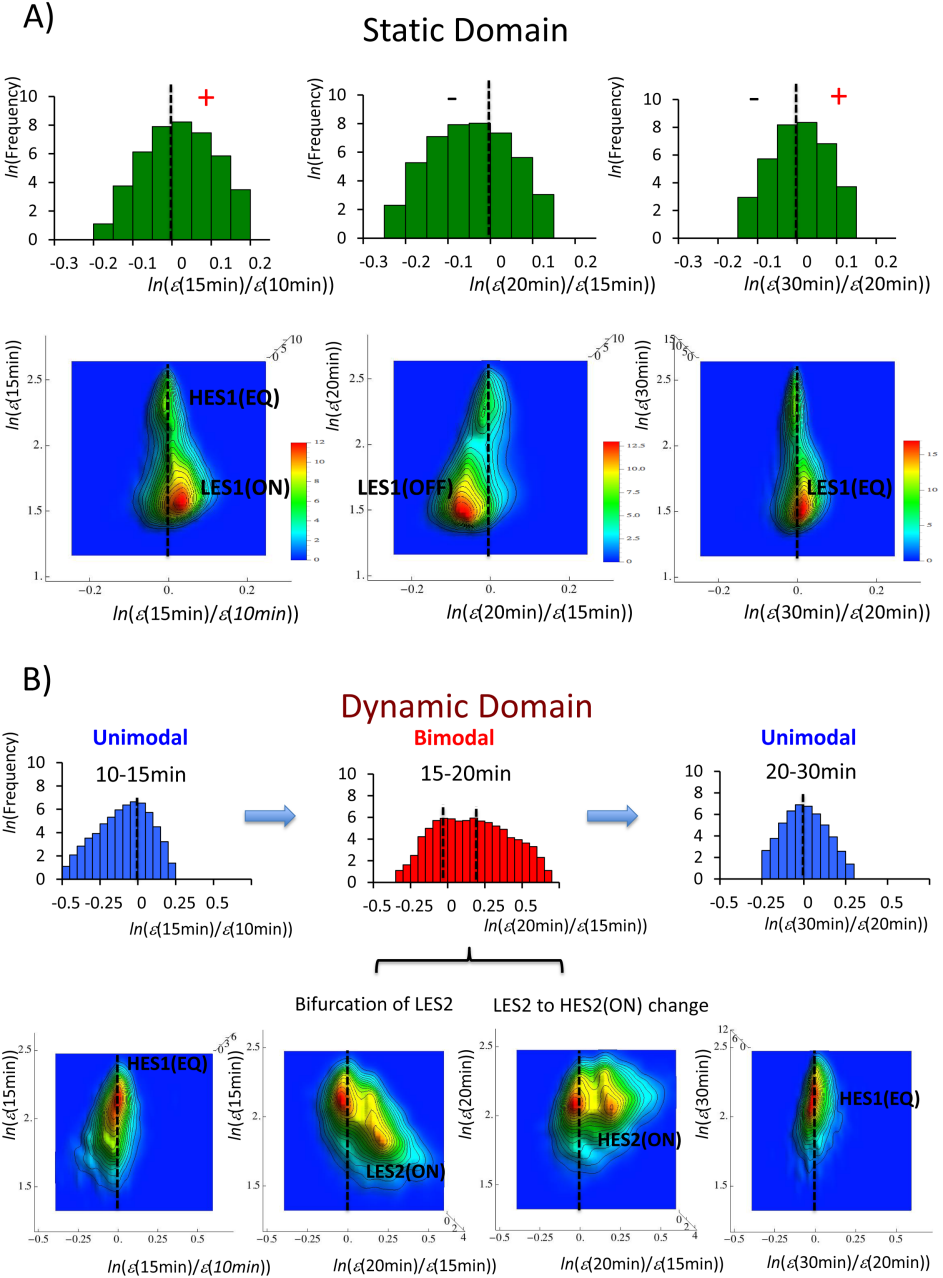
Dynamic motion of the characteristic HRG domains in DEAB of the expression change. The coordinated expression dynamics around an equilibrated high-expression state exhibit the pendulum oscillation of CES (autonomous bistable switch) between different time periods (10–15 min, 15 min–20 min, and 20–30 min): A) in the static domain (9059 mRNAs) LES1 shows ON-OFF-EQ oscillation around HES1(EQ) through a unimodal shift, and B) in the dynamic domain (3269 mRNAs) the bifurcation of LES2 at 15 min shows a dynamic change from LES2(ON) to HES2(ON) through a unimodal to bimodal profile at 20 min, and the annihilation of HES2 through a bimodal to unimodal profile at 20–30 min around HES2(EQ). The annihilation of HES2 reveals a short-lived dynamic domain. First row: frequency distribution of the expression change. Second row: the genetic landscapes of A) the static domain and B) the dynamic domain from the top view with density color bars.

The time-dependent change in the characteristic domains on the landscape with HRG (Figures 7A–B) exhibited temporal change and the bifurcation of CES, whereas the EGF domains did not show any apparent change (data not shown), as revealed by DEABs (Figures 1A–B). In the static domain for HRG, the low-expression state exhibits temporal change; LES1 is switched from ON (10–15 min) to OFF (15–20 min) (Figure 7A). The ON-OFF switch of LES1 is consistent with the change in DEAB from near-equilibrated to down-regulated for the HRG static domain (*rmsf* <0.21) between 10–15 min and 15–20 min (Figure 1B).

Figure 7B shows a pair of CESs forming a pendulum of oscillation, in which the pendulum of one CES oscillates around another CES: the low-expression state, LES1_HRG_, swings around the high expression state, HES1_HRG_(EQ), in the HRG static domain, where the EQ level corresponds to the lowest energy level of the pendulum system. In the dynamic domain, the pendulum motion is accompanied by bifurcation and the annihilation of CES (Figure 7B); a CES oscillates around HES1_HRG_(EQ). Three are elicited during the period 15–30 min: the bifurcation of LES2_HRG_ at 15 min, the change from LES2_HRG_(ON) at 15 min to HES2_HRG_(ON) at 20 min (unimodal shift: Figure 6A), and the annihilation of HES2_HRG_ at 20–30 min. Thus, the annihilation of HES2 reveals that the HRG dynamic domain is short-lived; the characteristics of the domains vary with time through dynamic bifurcation. The pendulum of oscillation based on a pair of CESs shows *autonomous bistable switch* (ABS) as shown in Figure 9, in order to represent the scenario in the dynamic bifurcation in a schematic manner without the usage of any mathematical equation. The dynamics of characteristic expression domains exhibits non-equilibrium dynamics through the energy flow between the oscillations of CES.

**Figure 8:**
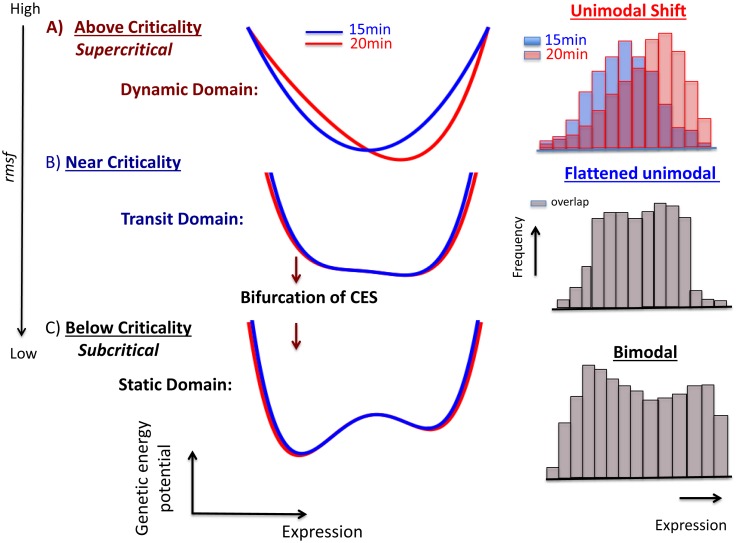
Schematic illustration of genetic criticality in DEAB of the expression. The putative genetic energy potential (15 min: blue; 20 min: red) with a fixed change in the expression (see the main text) describes the arrangement of mRNA expression in a transcriptional system, where the profile of the potential is anticipated from the scaling exponent of the frequency distribution of the expression (histograms: right panel; blue: 15 min; red: 20 min; gray: overlapped). The potential profile follows a change from single-well to double-well through a flattening profile as the *rmsf* is decreased (black arrow). The picture exhibits genetic criticality (details in Table 1 and File S1) as interpreted by the Landau theory [28] on characteristic expression domains for the HRG genomic response: dynamic (dark red), transit (dark blue), and static (black) domains represent above-, near- and below-criticality, respectively. In the above-criticality, due to the unimodal to bimodal shift of the frequency distribution (see also Figure 6A), the energy potential should also be shifted; in the near-criticality and below-criticality due to the overlapping frequency distributions between 15 min and 20 min, the energy potential should be (almost) temporally invariant. Note that, in the double-well potential (below-criticality), instead of generating two independent Boltzmann distributions (two equilibrium states), the frequency distribution shows broken distributions, which suggests non-linear interaction between coherent expression states in a non-equilibrium system.

**Figure 9:**
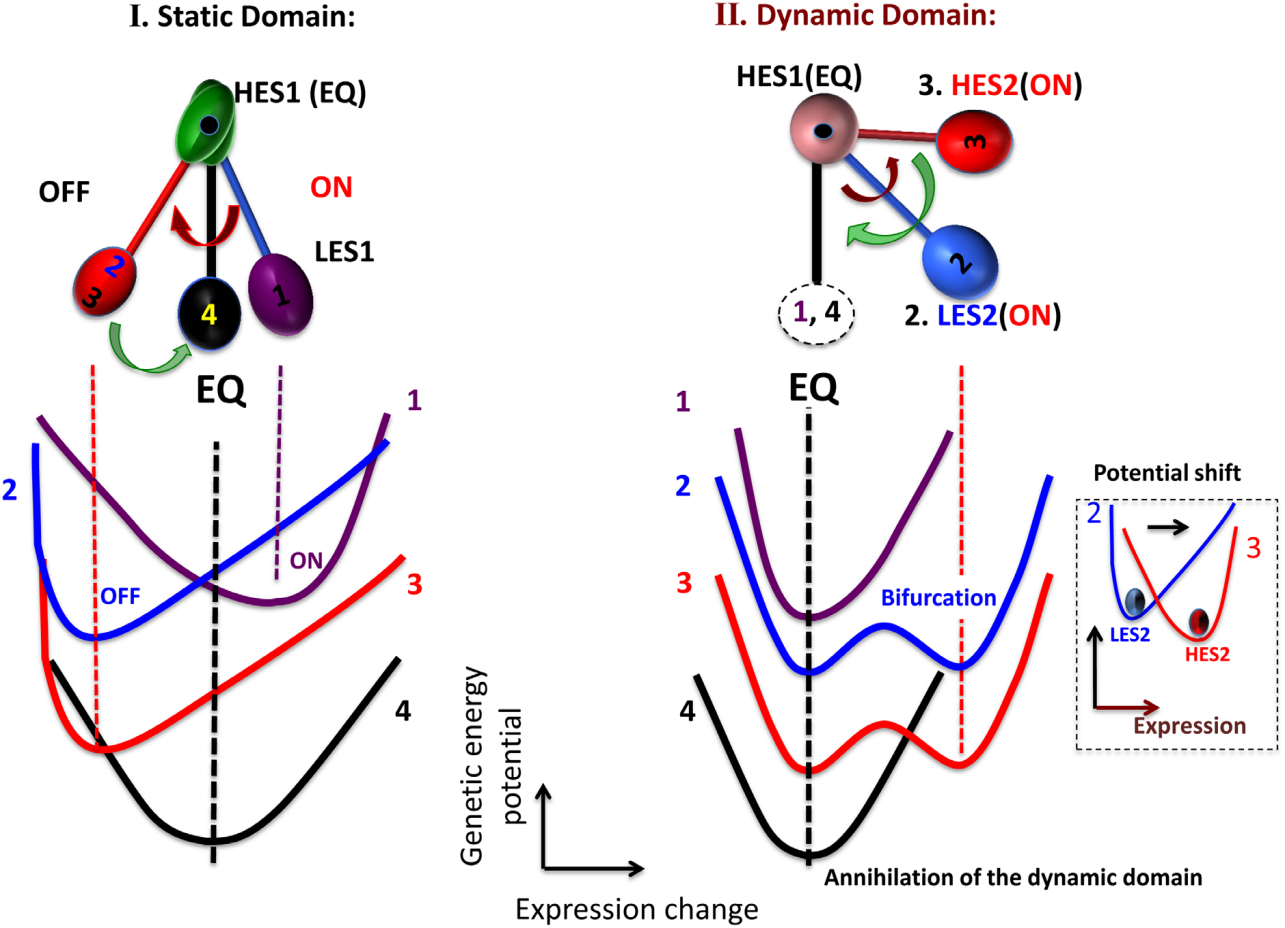
Schematic illustration of autonomous bistable switch (ABS) with genetic ‘energy profile’ in DEAB of the expression change. First row: the schematic illustration depicts the temporal development of ABS showing the opposite changes of pendulum oscillation of CES between the static and dynamic domains (refer to Figure 7). In the HRG static domain (left panel), the temporal change of CES (LES1) occurs without the bifurcation of CES; in the dynamic domain (right), the pendulum oscillation occurs through the dynamic bifurcation of CES: bifurcation of a low-expression state with a change in a putative potential profile from single- to double-well at 15 min, a change from the low- to the high-expression state at 20 min (refer to Figure 6A) with a single-well potential shift (small dashed box), and annihilation of the high-expression state with a change from double- to single-well at 20–30 min. Second row: schematic illustration describes the dynamics of the genetic energy potential as a function of the expression change (with a fixed expression; see details in the main text): 1. purple line: 10–15 min (at 15 min); 2. blue: 15–20 min (at 15 min); 3. red: 15–20 min (at 20 min); 4. black: 20–30 min (at 30 min). The picture shows the energy flow between the pendulum motions, which reflects the non-equilibrium dynamics of CES.

Regarding the bifurcation of CES associated with the change in the frequency profile for DEAB in the dynamic domain, interestingly, at 15–20 min, the frequency distribution of the expression for HRG shows a unimodal shift (Figures 6A and 8), while the frequency distribution of the expression change (Figures 7B and 9) shows a bimodal profile. Thus, in the dynamic domain, the bifurcation of CES (at 15 min) occurs in DEAB of the expression change, while the bifurcation of CES does not occur in DEAB of the expression at 15–20 min.

In summary, the temporal change of the low- to high-expression state (LES2_HRG_ to HES2_HRG_) around HES1_HRG_(EQ) explains the up-regulated motion of the dynamic domain on DEAB of the expression change, whereas the swing of LES1_HRG_ which induces an ON to OFF switch (opposite change for the dynamic domain) describes the down-regulation of the static domain. In the HRG transit domain, the high-expression state, HES1, showed the same pendulum swing as the static domain.

## Discussion

To understand the principles that underlie the global genetic response, as shown in Figures 1A–B, it is important that we shed light on how a CES, which is formed as a hill-like function on each domain, self-emerges on the genetic landscape. Gene expression is the result of complex biochemical reactions between genomic DNA and surrounding proteins following physicochemical laws (these laws do not imply that the expression process is deterministic, but rather this process is stochastic). As a central question, we address how coordinated gene expression that encompasses thousands of mRNAs is possible in a dynamic molecular transcriptional system from an understanding of the basic mechanism of self-organizing physical systems.

### Principle of the Self-organization of CESs Underlying the Genomic Response: Criticality of Self-organized Coherent Expression

According to the degree of *rmsf* (i.e., standard deviation of temporal fluctuation of mRNA expression), bifurcation of coherent expression of ensemble of mRNAs (coherent expression state: CES) clearly revealed three characteristic expression domains: dynamic, transit and static domains in cell differentiation (Figure 5: HRG). The corresponding frequency distribution of the ensemble of mRNA expression for each domain changes its profile from unimodal to bimodal through flattened unimodal profile (Figure 6A). Figure 4 showed that the onset of bimodal profile accompanies bifurcation of a CES.

The result indicates: 1) Power-law behavior defined by the order parameter *η*, such as *aη+bη^2^+cη* ^4^ merges near the critical condition, where a unimodal expression profile becomes flattened as discussed in more details in Supporting Information (File S1); a unimodal to bimodal change suggests that the variables of mRNA expression and temporal change in expression (Figures 4, 6 and 7) play the role as the important parameters, being something like the order parameter such as density to describe the coordinated expression behavior in terms of statistical physics. 2) Bifurcation of CES occurs from a unimodal onto a bimodal profile (Figures 6A and 7A); the peak of the frequency distribution coincides with the highest density of CES (Figure 4). Therefore, the result suggests that a unimodal to bimodal change in the frequency distribution might be related to change in stability of the profile that causes the bifurcation of CES.

A well-known example of critical behavior is the spontaneous ferromagnetization of iron through the synchronization of spins of the components with temperature; below the critical temperature (*T*<*T*_c_: subcritical), the system is ferromagnetic (long-range ordered) and above it (*T*>*T*_c_: supercritical), the system is paramagnetic (disordered). Unlike the spins of ferromagnetic iron, the ensemble of gene expression does not behave in unison; rather, stochastic gene expression was seen around the mean value of the group in DEABs based on the grouping of *rmsf*, as shown in Figures 1A–B. Interestingly, recent theoretical and empirical results of emergent neural collective phenomena in the brain support the concept that ‘the brain is naturally poised near criticality’ [32].

The occurrence of a unimodal to bimodal frequency change in expression profile of the ensemble of mRNAs through the bifurcation of CES suggests evidence of the genetic transition that possesses features of criticality. The mean field behavior of bimodal critical phenomena can be interpreted in terms of Landau theory [28]. The significance of the potential occurrence of ‘genetic criticality’ in DEAB of the expression provides an underlying principle for how genome-wide genetic activities are self-organized. Thus, criticality can be a fundamental biophysical mechanism in regard to how a cell with a small, compact nucleus space can conduct robust control of genome-wide coordinated gene expression for a short time (in our case within 5 minutes), despite of the underlying stochastic nature and heterogeneous environment.

We discuss the details of the genetic criticality in terms of Landau’s approach in the Supporting Information (File S1). In brief, the frequency distribution of the expression (natural logarithm of mRNA expression: scaling exponent) follows a unimodal to bimodal change (Figures 6A–B) that is accompanied by the bifurcation of CESs. Thus, the profile of the negative scaling exponent reflects the profile of the ‘genetic energy potential’, i.e., the energy of the transcriptional system to arrange expression. The genetic energy potential follows a change from a single-well to a double-well function like a Mexican hat. The genetic energy potential is a function of at least two variables: mRNA expression and its temporal change in expression, so that the genetic energy potential for expression (or change in expression) is obtained under a fixed change in expression (or expression).

We noted that the bifurcation of CESs in DEAB of the expression for HRG self-organizes genome-wide expression through genetic criticality (Table 1 and Figure 8): the dynamic domain (high *rmsf*: *rmsf* >0.42): *above criticality* (supercritical) with the unimodal frequency distribution that reflects the existence of a single-well energy potential, the transit domain (intermediate *rmsf*: 0.21< *rmsf <*0.42): *near criticality* (critical) with broadening of the unimodal profile (i.e., broadening of a single-well energy potential), and the static domain (low *rmsf*: *rmsf <*0.21): *below criticality* (subcritical) with the bimodal distribution (reflects the existence of a double-well energy potential). The EGF response does not show above-criticality; which suggests that the genomic state induced by EGF could be a ‘transit’ genomic state, which is ready to shift to be either up- (ON) or down-regulated (OFF).

With regard to DEAB of the expression, genetic criticality is expected (Figure 8), whereas with regard to DEAB of the expression change, CES dynamics, i.e., ABS to explain the temporal development of criticality (dynamic criticality; Figure 9 and File S1) was revealed. Thus, if we combine the pictures of the dynamics of CES for both DEABs, coherent gene-expression dynamics are reflected in the genetic energy potential as a function of mRNA expression and the temporal change in expression (see details in File S1). Notably, with regard to the HRG dynamic domain, the key domain that caused a biphasic response was annihilated at 20–30 min, which showed that ABS without HES2 is back to an equilibrated state (Figure 9) through the bimodal to unimodal transition.

To help clarify the unique time-dependent genetic behavior found in the present study, in Figure 9 we illustrated ABS with a time-dependent (dynamic) energy-like potential as expected from the scaling exponent of the frequency distribution of the expression change (Figures 7A–B). The bifurcation of CES induced by the energy flow between oscillations of CES is clearly depicted. In contrast, the ON-OFF switch of LES1 occurred through a unimodal shift (Figure 7A) as well as a dynamic change from a low- to a high-expression state (LES2 to HES2: Figure 6A). This suggests that the stability change in the profile (i.e., unimodal to bimodal transition in frequency distribution, and vice versa) does not occur for either an ON-OFF switch or for the change from a low- to high-expression state.

### Higher-order Structural Transition of DNA as a Possible Biophysical Origin of Genetic Criticality

We observed coordinated genome-wide mRNA expression dynamics and anticipate the presence of genetic criticality in the genome-wide coherent mRNA expression dynamics. However, the biophysical origin of such genetic criticality is not clear.

Gene expression in a mammalian cell involves the dynamic read-out of genetic information on a specific genomic DNA sequence, which is tightly regulated by chromatin folding/unfolding dynamics; chromatin can exist in an open form (loosely packed), which allows the transcriptional machinery to bind a specific genomic DNA sequence, or a closed form (densely compacted), which makes DNA inaccessible, in the presence of chromatin remodeling proteins, thus determining which genes are expressed or not [33]–[35].

It has been well established that DNA molecules undergo on/off transition between swelled state and compact state on the scale of 100 kbp–1 Mbp accompanied by a change in density on the order of 10^4^–10^5^ [36]. Thus, the elongated swelled state of DNA can induce ON expression, whereas the compact state turns expression off, i.e., OFF expression. The time-scale of this conformational transition is rather fast, on the order of less than minutes according to the *in vitro* observation, which is consistent with the biphasic genomic response. Such discrete transition is induced by the change of environmental parameters, such as the concentration of RNA, polyamine, NTP, magnesium ion, etc [37], [38].

On the other hand, specific key-lock interaction through transcriptional machinery is well known to cause the change on DNA only up to the order of kbp. Thus, it may be of natural that change on the environmental parameters in nucleus may cause on/off type transition simultaneously on many domains in the whole genome; although such hypothesis is only in an immature state, a plausible scenario might be that through signaling transduction, the control of production of RNA molecules, that are able to induce the switch between the compact and elongated states of local DNA [38], leads to the induction of the transition of specific regions of genomic DNA; this in turn might coordinate the initiation of coherent genomic DNA transitions to provoke a global genetic response.

The crucial elucidation might be to decipher intra- and inter-chromosome interaction for each characteristic domain to grasp coherent expression dynamics, especially to understand the relationship between ABS in the static domain (Figure 7A) and genomic DNA higher-order structural transitions. We expect that the temporal change of LES1_HRG_ (coordinated low expression) in the static domain could result from the coherent activity of genomic DNA higher-order structural transitions through non-specific interactions between genomic DNA and environmental factors, where mainly low expression dynamics are anticipated, while the remaining HES1_HRG_ (coordinated high expression) in an equilibrated state reflects a stable swelled state of the ensemble of genomic DNAs. Furthermore, understanding of the opposite pendulum motions seen in the static and dynamic domains (Figures 7A–B and 8) for a short time period might provide important insight into the interactions among conformal DNA transitions, such as cross-talk [39].

Along these lines, the coordinated relationship between key-lock molecular transcriptionally machinery and the genomic DNA on/off transition in gene expression dynamics could be revealed, such that the organized activity of the molecular transcriptional machinery on coiled/swollen genetic DNAs regulates coherent mRNA expression dynamics in the dynamic domain (with a unimodal expression profile), whereas in the static domain (with a bimodal profile), coherent genomic DNA on/off transitions predominate in the dynamic control of coherent gene expression through the balanced stage in the transit domain. Thus, it is reasonable to expect that the characteristic expression domains based on the standard deviation of temporal expression fluctuation (i.e., *rmsf*) should be related to the plasticity of genomic DNA: a higher *rmsf* is associated with a more pliable DNA structure; interestingly, the conformational transition of DNA, such as an all-or-none discrete transition [40]–[42], stepwise collapse or continuous gradual change [39], [43], [44], is induced depending on “nonspecific” interactions between a DNA molecule and environmental factors within a cell nucleus.

Finally, it is important to point out that while the bifurcation of CES occurs through a unimodal to bimodal frequency change, the dynamic motion of CES is induced by a unimodal shift, which suggests that genetic criticality might result from a continuous phase transition of genomic DNA, where stochastic expression except for experimental noise stems from unspecific interaction between genomic DNA and environmental factors. Hereafter, we might examine whether our hypothesis stands on the underlying mechanism of the control of whole genome.

## Conclusion

We examined how it is possible for differentiating MCF-7 breast cancer cells to carry out the robust self-control of dynamic genome-wide gene expression compared with that in the proliferating state. Through the grouping of mRNA expression, dynamic ensemble averaging behaviors (DEABs) of gene expression and differences in gene expression over time: DEAB of the expression (Figure 1A) and the expression change (Figure 1B), respectively, were shown to reflect the existence of genome-wide coordinating expression dynamics, which clearly differentiated between the HRG and EGF genomic responses in a biphasic manner, i.e., all-or-none response (Figures 1A–B). The coordinating expression dynamics stemmed from the dynamics of a coherent expression state (CES) to form a hill-like function on the genetic landscape (Figure 3). The different coordinated genome-wide expression dynamics in cell proliferation (EGF) and differentiation (HRG) were revealed in DEABs of the expression and the expression change:

1) In DEAB of the expression, a coordinated motion of the ensemble of mRNA expression emerges as a CES by analyzing expression dynamics between different time points. The dynamic ensemble motion of DEAB of the expression with a change in the slope from negative (15 min) to positive (20 min: Figure 1A) was explained by the change from low-expression state (LES: 15 min) to high-expression state (HES: 20 min) with a unimodal shift in the dynamic domain (Figure 6A). The scenario of bifurcation of CES (Figure 5) at 15–20 min revealed distinct characteristic expression domains between the HRG and EGF responses (Figures 6A–B and 8; Table 1): dynamic, transit and static domains in the HRG response, but only transit and static domains in the EGF response.

The scenario of bifurcation of CES in DEAB of the expression exhibited the presence of criticality in expression profile as interpreted by Landau’s theory (Figure 8 and Table 1): a unimodal (dynamic) to bimodal (static) change through flattening of the unimodal profile (transit domain) in the HRG response with a decrease in *rmsf*, whereas in the EGF response, the bifurcation of CES occurred only through flattening of a unimodal to bimodal profile. As an underlying principle, criticality in genetic dynamics self-organizes CESs into characteristic domains that are distinctively different between the HRG and EGF genomic responses for a route to genomic transition. To grasp the essence on global genetic dynamics, it might be useful to interpret the bifurcation of CES on the profile, from unimodal into bimodal, in terms of “symmetry” and “symmetry breaking” [30] (see more discussion in File S1).

2) In DEAB of the expression change, the coordinated expression dynamics emerges as autonomous bistable switch (ABS) - the pendulum oscillation formed by a pair of CESs - between different time periods (10–15 min, 15–20 min and 20–30 min). ABS in the static domains at 15–20 min described the all-or-none response: the pendulum motion of the low-expression state from up- (at 15 min) to down-regulation (at 20 min) around the high-expression state in the HRG static domain, which was opposite motion in the HRG dynamic domain (Figures 7 and 9), while there was no apparent change in the EGF response (clearly shown in Figures 1A–B).

The scenario of dynamic bifurcation suggested the temporal development of criticality (dynamic criticality: Figure 9): in the dynamic domain, the bifurcated CES (Figure 7B) at 15 min changed from LES to HES at 20 min (unimodal shift: Figures 6A and 7B) in the pendulum swing, and annihilated at 20–30 min, which revealed a short-lived dynamic domain.

Thus, molecular biological follow-up associated with epigenetic modifications for the dynamic domain might reveal crucial biological processes that determine the early cell-fate decision in MCF-7 cells. Furthermore, the short-lived dynamic domain implies that genes of relatively small fluctuation in the transit and static domains may play the role as a foundation of the global genetic response, which is consistent with the indication in our recent works concerning the major roles of low variant genes in global collective modes [Tsuchiya M et al., unpublished].

We expect that the characteristic expression domains associated with the dynamics of CES should be related to distinct genomic structures that result from coherent epigenomic activity such as methylation and acetylation that are accompanied by a steric change in genomic DNA with a size of several kbp to several Mbp. We discussed the anticipated potential biophysical origin of genetic criticality (the co-existence of above-, near- and below-criticality) in terms of the genomic DNA phase transition. Based on the genomic structure [45]–[48], an analysis of characteristic domains on chromosomes may help to elucidate the dynamic interaction in chromatin-folding or -unfolding dynamics [49] through the genomic DNA structural transition. This interaction may represent a basic mechanism for the induction of a global genetic response as well as a mechanism for systemic genomic control in cell differentiation.

## Methods

### Biological Datasets

We analyzed time-series Affymetrix GeneChip (Affymetrix U133A 2.0 chip) microarray data (Gene Expression Omnibus database ID: GSE13009) that included all human gene expressions in MCF-7 breast cancer cell line under the addition of distinct ErbB receptor ligand, either EGF or HRG-β [50]. We evaluated expression levels of 22035 Affymetrix probe set IDs and normalized data using Robust Multichip Average (RMA) for further background adjustment and the reduction of false positives [51]–[53]. In the microarray experiment, there are two repeated data (rep 1 and rep 2), where rep 1 was analyzed in this report and the bifurcation of CES (Figure 5) for rep 2 was shown in File S2 (Supporting Information). The complete experimental details were reported by Saeki et al. [50].

### Grouping of mRNA Expression

In studies of large-scale, high-throughput gene expression, such as in microarray experiments, it can be difficult to estimate the signal intensity. Especially, in microarray experimental data, expression noise stems from two major sources [54]–[56]: biological noise due to asynchronous cell activities in cell culture, which results in the fluctuation of mRNA production from average values of gene expression, and experimental noise, which can arise from the array material, estimation of the amount of mRNA, unspecific binding between probes and target mRNAs, etc. Most expression noise is considered to be unspecific and uncorrelated. In low-level expression, the relative effect of measurement noise, compared with the specific binding activity, is expected to be much larger than that for highly variable genes. Thus, in many studies, a subjective expression threshold was introduced to cut off the majority of gene expression, which might not accurately reflect the correlation among mRNA expression if such a global correlation exists [18]–[20].

To examine genome-wide expression dynamics, all mRNA expression (*N* = 22035) in MCF-7 cells was sorted from the highest temporal fluctuation to the lowest, and the root mean square fluctuation (*rmsf*) of expression for each mRNA was evaluated at 18 time points:



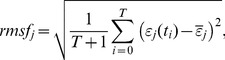
(1)where *rmsf_j_* is *rmsf* of the *j^th^* mRNA, which has the expression, *ε_j_*(*t_i_*), at *t* = *t_i_* (*i* = 0,..,17; *j* = 1,..,*N*); and 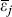 is the average expression value of the *j^th^* mRNA over the 18 time points: *t*_0_ = 0, *t*_1_ = 10 min, 15, 20, 30, 45, 60, 90 min, 2 h, 3, 4, 6, 8, 12, 24, 36, 48, *t_T_*_ = 17_ = 72 h. Note that *rmsf_j_* is not time-dependent, and that a low (high) *rmsf* value does not mean that the expression is low (high); i.e., temporal fluctuation from the average expression is small (large). Next, we divided the sorted genes into *k* groups with an equal number *n* of mRNAs in the genome (*k* = *N/n*), where *k* is integer of *N/n*; *n* is the number of mRNAs. Ensemble averages <*rmsf* > and <*ε*> are defined as the simple arithmetic mean over an ensemble or a group of mRNAs.

We sorted mRNA expression according to the value of *rmsf* over all time points and investigated the genomic response at 15–20 min. It is important to note the possible discrepancy in the regulation of expression between the standard deviation of the change in expression (Eq. 1) and the change in expression if we only consider from 15 min to 20 min; for instance, when we compared 3269 mRNA expressions in the dynamic domain of HRG with the same number of the most up-regulated expressions from *t* = 15 to *t* = 20 min, we found that only about 57% of the mRNAs (1878 probes) were common between them. Biphasic statistics emerged from the grouping based on *rmsf*, and thus the analysis of these two ensembles of mRNAs may lead to different models (or modes) of gene regulation.

## Supporting Information

File S1 **Dynamic Emergent Averaging Behaviors (DEABs) through the grouping of genes and Dynamic Criticality with Figure S1.**
(PDF)

File S2 **2D plots of genetic landscape to show the bifurcation of coherent expression states in DEAB of the expression for HRG and EGF for repeated experimental data (rep 1 and rep 2).**
(PDF)
