# Correction: Global Genetic Response in a Cancer Cell: Self-Organized Coherent Expression Dynamics

**DOI:** 10.1371/journal.pone.0105491

**Published:** 2014-08-08

**Authors:** The PLOS ONE Staff

There are errors in the header of Table 1 due to errors that occurred in the typesetting process. The correct version of Table 1 can be viewed below. The publisher apologizes for this error.

**Table 1:** Bifurcations of CES, Characteristic Domains and Criticality at 15-20min.

	HRG			EGF	
Criticality	Above	Near	Below	Near	Below
Domain	Dynamic	Transit	Static	Transit	Static
Range of *rmsf*	>0.42	0.21–0.42	<0.21	>0.16	<0.16
# of mRNAs	3269	9707	9059	14944	7091
Distribution: mRNA expression	Unimodal	Flattened Unimodal	Bimodal	Flattened Unimodal	Bimodal
DEAB of the expression	Up	No change	No change	No change	No change
Coherent Expression State: CES	HES1 (EQ) and HES2 (ON)	HES1 (EQ)	HES1 (EQ) and LES1 (OFF)	HES (EQ)	HES (EQ) and LES (OFF)
Bifurcation of CES	LES2 (ON)	Onset of birth of LES1	LES1 (OFF)	Onset of birth of LES	LES (OFF)
State change	LES2 (ON;15min) → HES2 (ON;20min)	-	-	**-**	-
