# Supplementary material for: Global Genetic Response in a Cancer Cell: Self-Organized Coherent Expression Dynamics
Source: PLoS One. 2014 May 15;9(5):e97411. doi: 10.1371/journal.pone.0097411 (PMC4022610; doi:10.1371/journal.pone.0097411)
Supplement: File S1 — Dynamic Emergent Averaging Behaviors (DEABs) through the grouping of genes and Dynamic Criticality with Figure S1. (PDF) [file pone.0097411.s001.pdf]

Supporting Information S1

Dynamic emergent averaging behaviors (DEABs) through the grouping of genes

The grouping of gene expression based on the degree of temporal change in expression (e.g., *rmsf*: see the Method) provides us different views of the same system at different ‘magnification scales’ or course graining, which corresponds to the different sizes of selected groups. Initially, we considered the grouping of genes might reduce noise due to the ‘unspecific’ nature of expression noise in microarray data, which contains biological and experimental noise [1-3]. In our prior works, for LPS innate responses of macrophages and naive CD4+ T cell differentiation with a few experimental time points, groups are made according to changes in expression with respect to initial (at time t_0) wild-type genome, whereas for HL-60 cell (human promyelocytic leukemia cell) differentiation with enough experimental time points like MCF-7 cell in this report, grouping is based on an expression variation, where average value of each gene expression in time is subtracted from gene expression, and genes are sorted by the standard deviation of expression in time. In any case, groups are based on pure expression level basis with no reference to the physiological role of the genes.

We found that the average value ($\bar{x}_i$) of the ith group between different time points or different genotypes ($\bar{y}_i$) exhibited nonlinear correlation (curve) between $\bar{x}_i$ and $\bar{y}_i$ as the group size *n* (the number of probes in each group) increased: $\bar{y}_i = f(\bar{x}_i)$ [4-6]. Interestingly, the emergence of global trend (manifold) from scattered points to (asymptotic) time-dependent correlation was noticed in the distribution of the whole groups when the grouping size is about *n* = 30-50. Especially, as the group size (*n*) increased, the ordinary square root distance (Euclidian distance) from the correlation decreases based on the inverse square root law ($\alpha/\sqrt{n}$: α is a constant coefficient); the average value of a group approaches to an asymptotic value, which indicates a distinct

asymptotic frequency distribution for temporal expression of genes within the group [5]. We progressively approach to the 'true' (i.e., asymptotic) distribution relative to the reference population, in which each gene expression in a group is stochastically fluctuating around the asymptotic average value. Such global trend on the whole gene expression imply the presence of a hidden governing principle, which rationalizes groups of genes to guide all the genes allowing for such a convergent behavior when the entire expression set is sampled [Tsuchiya M, Hashimoto M, Tomita M, Yoshikawa K, Giuliani A, "Collective Genome-Wide Expression Modes: Major Roles of Low-Variance Genes", unpublished].

Notably, as the biological and biophysical significance, i) for the innate immune response induced by lipopolysaccharide (LPS) stimulus to Toll-like receptor 4, we demonstrated the first time to reveal local and global effects of LPS on the genomic response of macrophages in wild-type and mutant conditions; the local response reflects the well-known proinflammatory response of a small number of high-variance genes (about hundreds), while the global response reflects the novel collective/coordinated activation of diverse processes comprising the rest of majority number of low-variance genes. The global property of the immune response emerged as a result of the transition from large scattered expression for a single gene to smooth linear correlations for grouped genes that can be linearly superposed to decipher the global gene regulatory differential control of the transcriptional and mRNA decay machineries between wild-type and mutant genomes [4]; ii) for T-cell differentiation, we observed global switching behavior of gene expression between help T1 and T2 differentiations [5]; iii) for HL-60 cell differentiation, gene expression dynamics exhibits coherent oscillating behaviors during differentiation process [6]. Furthermore, we demonstrated, in a statistically rigorous manner, the existence of specific gene ensembles (collectively named "genome vehicle") responsible for the formation of a neutrophil attractor; the collective motion of lowly and moderately variable genes within a genome vehicle guides the whole gene expression toward the neutrophil attractor.

These results revealed the emergent hidden global regulations in genome-wide expression, which drives orchestrated diverse regulations of biological processes. Thus, there must be organized principles to explain such highly coordinated averaging

responses of biological processes, which might imply the existence of simple rules that control and regulate complex biological processes. Next, we address a basic underlying mechanism to derive the global genetic response.

Dynamic Criticality

We demonstrated that a change from a unimodal to a bimodal frequency distribution, or vice versa for both of the expression (logarithm of mRNA expression: $\ln(\varepsilon(t))$) and the change in the expression showed symmetry breaking in dynamics of coherent gene expression. This resulted in the good coincidence of peaks between density of coherent expression states (CESs) and frequency distributions, which suggests the existence of energy like potential that encompasses thousands of mRNA transcriptional reactions. The dynamics of CES with a unimodal shift further supports dynamics of energy like potential (i.e., the energy flow; examples in Figures 8 and 9).

Most interestingly, the bifurcation scenario of CES in DEAB of the expression (Figure 5) showed the distinct characteristic expression domains for biphasic statistics, and self-organized CESs exhibits 'genetic criticality' (Table 1 and Figure 8) in the expression profile: the underlying principle of the global genetic response. This can be a fundamental biophysical mechanism in regard to how a cell with a small, compact nucleus space can conduct robust control of genome-wide coordinated gene expression for a short time, despite of the underlying stochastic nature and heterogeneous environment. Here, we depict details of symmetry breaking in genetic criticality.

The physical phenomenon called symmetry breaking can be phenomenologically interpreted by Landau's symmetry argument [7-9]; the mean-field theory (averaging behavior) describes critical behavior through symmetry breaking. DEAB of the expression (Figure 1A) expresses mean-field behaviors of mRNA expression; the unimodal to bimodal symmetry breaking in the frequency distribution [10] of the expression according to DEAB (Figures 4 and 6) anticipates the occurrence of criticality, which encompasses genome-wide coherent expression. In genetic criticality, the expression possesses a characteristic of an order parameter such as density, and the parameter *rmsf*, which describes the standard deviation of temporal fluctuation in mRNA expression, depicts the

degree of symmetry breaking such as temperature to categorize critical phenomena as *above-criticality* (supercritical), *near-criticality*, and *below-criticality* (subcritical) (Figure 8); a local minimum of energy potential corresponds to a hilltop of a CES and a peak of the frequency distribution of the expression (Figures 6A-B).

We considered gene expression near criticality, where the potential energy (or energy like function) $F(\eta)$ could be expanded by the lowest order of the order parameter η: $F(\eta) = a\eta + b\eta^2 + c\eta^4$ with the linear external field, $a\eta$, where $\eta \doteqdot \ln(\varepsilon(t)) - \eta_0$ and η_0 is the expression value of the valley, which separates two phases (low- and high-expression states) of CES in the static domain (i.e., η_0= 2.075). As shown in the schematic illustration (Figure S1), we depict the concept of symmetry breaking for a *near equilibrium system*: A) in above-criticality ($b\eta^2$: $a \approx 0$, $b > 0$ and $c = 0$; Figure S1 A), the dynamics of ensemble of molecules is governed by Brownian dynamics (Gaussian dynamics) in terms of the order parameter η (i.e., the expression), B) in near-criticality (small negative b and $c > 0$; Figure S1 B), the onset of symmetric-symmetry breaking (a = 0) from a unimodal to a bimodal state transition (i.e., a single local minimum to a double local minima in the energy potential) occurs at the critical point ($\eta = 0$), and C) in below-criticality ($b < 0$ and $c > 0$), the energy potential has the left-right symmetric local minima (Supplementary Figure S1 C) when the sign of the quadratic term changes from positive to negative: $b > 0 \rightarrow b < 0$ (from Figure S1 A to B) with a positive quartic term ($c > 0$). At around the near critical point (η = 0), the negative quadratic term $b\eta^2$ dominates the energy function to make it negative, and then away from the critical point, the positive quartic term $c\eta^4$ takes over the quadratic term to create double minima like a Mexican hat. At the symmetric-symmetry breaking, the whole system can move between two new states with an equal transition probability to produce two equivalent Boltzmann distributions. To make asymmetric-symmetry breaking ($a \neq 0$), the linear term $a\eta$ such as a role of electric field contributes to left ($a > 0$) or right side ($a < 0$) asymmetric local minima (Figure S1 C).

To understand a *non-equilibrium* process in genetic activity, we point to an example that the bimodal frequency distribution of the expression in the static domain (Figure 6) does not show two independent Boltzmann distributions as expected in a equilibrium system, where two peaks should correspond to peaks of a bimodal profile, but

rather the mixing of the expression, the broken Boltzmann distribution, appears (Figures 6 and 8). We expect that the mixing expression dynamics stems from nonlinear interactions among gene expression in a non-equilibrium system. In addition, the two high-expression states in the unimodal frequency distribution in the dynamic domain further support a non-equilibrium genetic activity (Figure 6A).

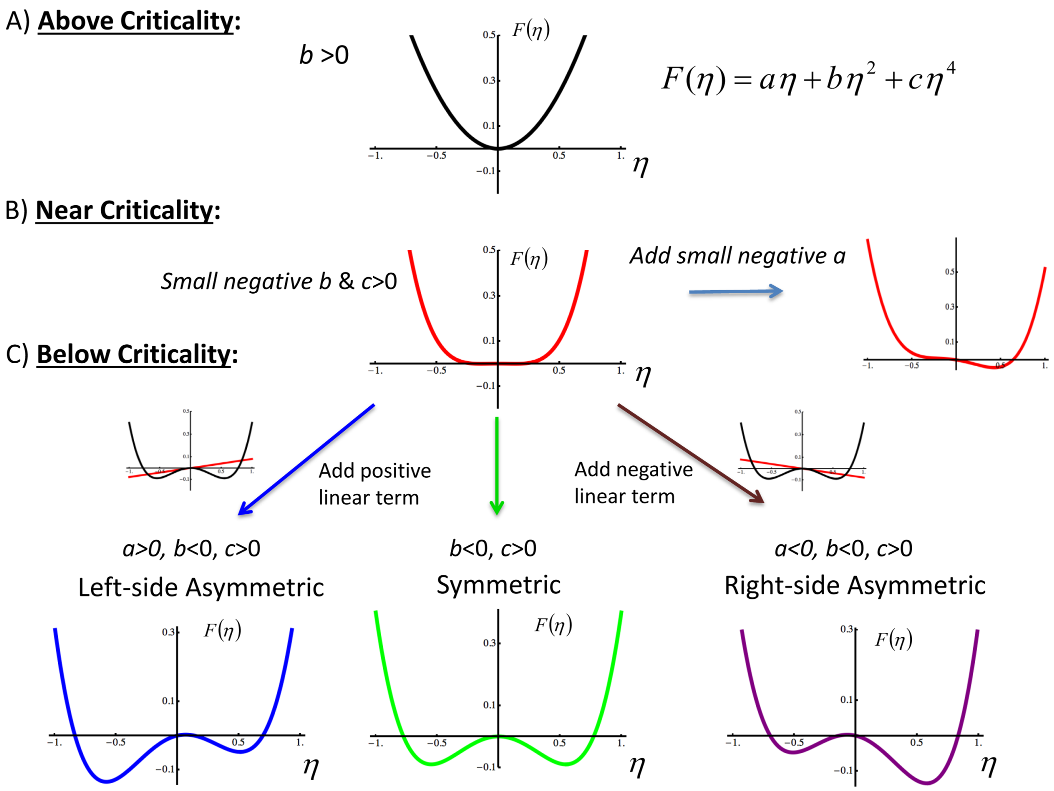

Figure S1: Criticality and Symmetry Breaking

Dynamic energy-potential, the time-dependent change in the energy profile is expected by the energy flow (due to a non-equilibrium process) in coherent expression dynamics (Figure 9). Self-organized CESs exhibit criticality in DEAB of the expression at a fixed time point (Figure 1A), while dynamics of a pair of CESs forms ABS (Autonomous Bistable Switch: a pendulum oscillatory system) to show the dynamic motion of a low-expression state in the HRG static domain and bifurcation and annihilation of a CES. In contrast, in the EGF response with no dynamic domain, as clearly shown in Figures 1A-B, pendulums in the static and transit domains remain in an

equilibrated state.

Combining the pictures of Figures 8 and 9, the time-dependent of criticality for the three domains in the HRG response with the symmetry argument is summarized:

i) In *above-criticality*: the dynamic domain, a single-well energy potential (profile) controls Brownian like gene expression, where transcriptional molecular (key-lock) activities expect to predominate coherent/coordinated gene expression dynamics on the ensemble of swollen genomic DNAs. The frequency distribution changes from a low-expression peak to a high-expression peak (a unimodal shift during the period 15-20min) due to a marked activation of transcription of mRNA through complex epigenetic molecular assembly. Together with the symmetry breaking on DEAB of the expression change during the period 10-20min (Figures 7B and 9), the dynamics of CES in the dynamic domain clearly shows: *a*) change from a low- to a high-expression state (LES2(ON) → HES2(ON)) on DEAB of the expression (Figure 8) in a unimodal shift, *b*) the bifurcation of the low-expression state, LES2(ON) at 15 min through a unimodal (at 10min) to a bimodal symmetry breaking (at 15min) swinging to HES2(ON) as ABS on DEAB of the expression change (Figure 9), and c) the annihilation of HES2(ON) swinging from a high- to an equilibrated-state occurred through a bimodal to a unimodal symmetry breaking, which revealed a short-lived dynamic domain.

ii) In *near-criticality*: the transit domains, the onset of bifurcation of a low-expression state (LSE) from a high-expression state (HSE) occurred. In a single-well potential with a minimum at a high-expression level, a low-expression state is about to bifurcate at another minimum with a low expression level (i.e., $a < 0$, $b < 0$, and $c > 0$ in $F(\eta)$; Figure S1 B). Here a negative quadratic term ($b < 0$) plays an important role in symmetry breaking with a positive quartic term ($c > 0$), which makes the energy profile flat, and a negative linear term ($a < 0$) creates the asymmetric-symmetry breaking at right hand (high expression) side. Due to the onset of the bifurcation (LSE), the high-expression state HES1 was observed to swing (the same oscillation as the static domain) during the period 10-30min (data not shown).

iii) In *below-criticality*: the static domains, a local energy minimum bifurcates from a high energy minimum through symmetry breaking (a negative quadratic and a positive quartic term) of the energy potential, which results in the creation of a potential

barrier that separates coherent expression into high and low. Furthermore, at the right side a deeper well (valley) of the genetic energy profile indicated a key effect of a linear external term ($a > 0$). During the period 10-20min, the energy profile for DEAB of the expression (*rmsf* < 0.21 with a fixed change in the expression) should be temporally invariant (due to overlapping of thee DEABs at 10, 15, and 20 min: Figure 1A). On the other hand, DEAB of the expression change (i.e., a fixed expression) for HRG (*rmsf* < 0.21) changed from up- to down-regulation (Figure 1B) as a part of the pendulum oscillation of LES1 between 10-15min and 15-20min (Figure 9), which suggests the occurrence of a single-well shift of the energy potential during the period 10-15min (Figure 9).

Upon elucidation of coherent interactions of genomic DNA folding/unfolding dynamics, a basic mechanism of the formation of ABS by a pair of CESs could be deciphered, and the biophysical significance of the opposite pendulum oscillations of CES between the HRG dynamic and static domains (Figure 9) might be revealed.

Reference

1. Southern E, Mir K, Shchepinov M (1999) Molecular interactions on microarrays. Nat Genet 21: 5–9.
2. Tu Y, Stolovitzky G, Klein U (2002) Quantitative noise analysis for gene expression microarray experiments. Proc Natl Acad Sci USA 99: 14031–14036.
3. Zhang L, Miles MF, Aldape KD (2003) A model of molecular interactions on short oligonucleotide microarrays. Nat Biotechnol 21: 818–821.
4. Tsuchiya M, Piras V, Choi S, Akira S, Tomita M, et al. (2009) Emergent genome-wide control in wildtype and genetically mutated lipopolysaccharide-stimulated macrophages. PLOS One 4: e4905.
5. Tsuchiya M, Hashimoto M, Tomita M (2012) Robust global regulations of gene expression in biological processes: A major driver of cell fate decision revealed. Proceedings of the 2012 ICME, pp. 744–749.
6. Tsuchiya M, Piras V, Giuliani A, Tomita M, Selvarajoo K (2010) Collective

dynamics of specific gene ensembles crucial for neutrophil differentiation: the existence of genome vehicles revealed. PLOS One 5: e12116.
7. Takenaka Y, Nagahara H, Kitahata H, Yoshikawa K (2008) Large-scale on-off switching of genetic activity mediated by the folding-unfolding transition in a giant DNA molecule: An hypothesis. Phys Rev E 77: 031905.
8. Nagahara H, Yoshikawa K (2010) Large system in a small cell: A hypothetical pathway from a microscopic stochastic process towards robust genetic regulation. Chem Phys Lett 494: 88–94.
9. Landau LD (1937) On the theory of phase transitions. Zh Eksp Teor Fiz 7: 19–32.
10. Ryabov D, Gribskov M (2008) Spontaneous symmetry breaking in genome evolution. Nucleic Acids Res 36: 2756–2763.
