# Supplementary material for: Global Genetic Response in a Cancer Cell: Self-Organized Coherent Expression Dynamics
Source: PLoS One. 2014 May 15;9(5):e97411. doi: 10.1371/journal.pone.0097411 (PMC4022610; doi:10.1371/journal.pone.0097411)
Supplement: File S2 — 2D plots of genetic landscape to show the bifurcation of coherent expression states in DEAB of the expression for HRG and EGF for repeated experimental data (rep 1 and rep 2). (PDF) [file pone.0097411.s002.pdf]

Supporting Information S2

2D plots of genetic landscape for repeated experimental data

In this file, we provide 2D plots of genetic landscape to show the bifurcation of coherent expression states in DEAB of the expression for HRG and EGF (see details in Figure 5), where contour lines denote probability density (see Figure 5). There are two groups of plots for rep 1 (for Figure 5) and rep 2 data (for a repeated analysis) from Gene Expression Omnibus database ID: GSE13009: first row for Figure 5 (rep 1) and second row for a repeated data (rep 2); a repeated microarray experiment confirmed the characteristic domains for both HRG (cell differentiation) and EGF (cell proliferation) during the period 15-20min.

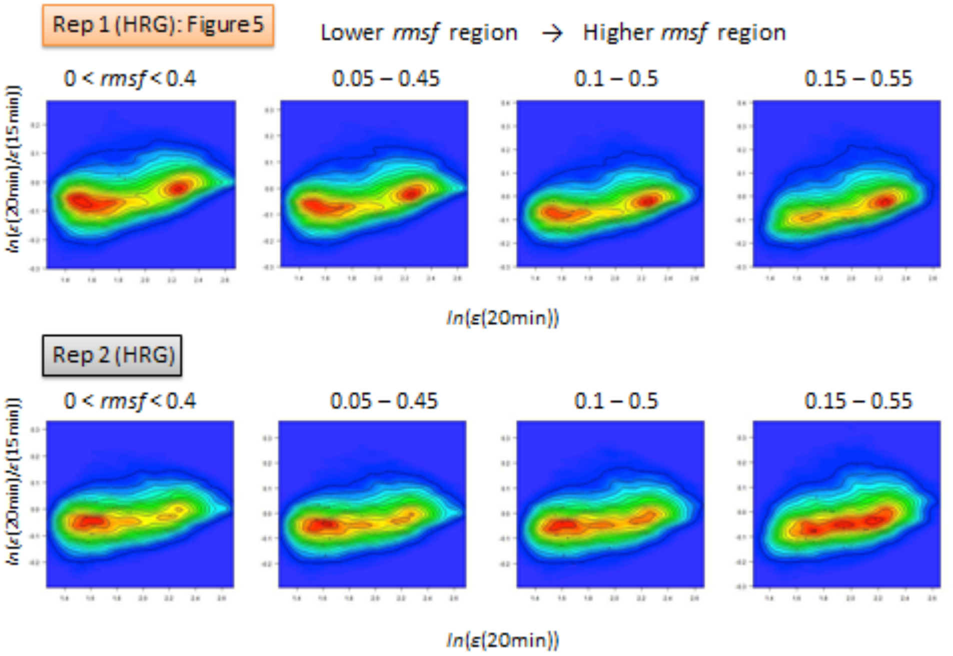
Rep 1 (HRG): Figure 5
Lower rmsf region → Higher rmsf region
0 < rmsf < 0.4
0.05 – 0.45
0.1 – 0.5
0.15 – 0.55
ln(ε(20min)/ε(15 min))
ln(ε(20min))
Rep 2 (HRG)
0 < rmsf < 0.4
0.05 – 0.45
0.1 – 0.5
0.15 – 0.55
ln(ε(20min)/ε(15 min))
ln(ε(20min))

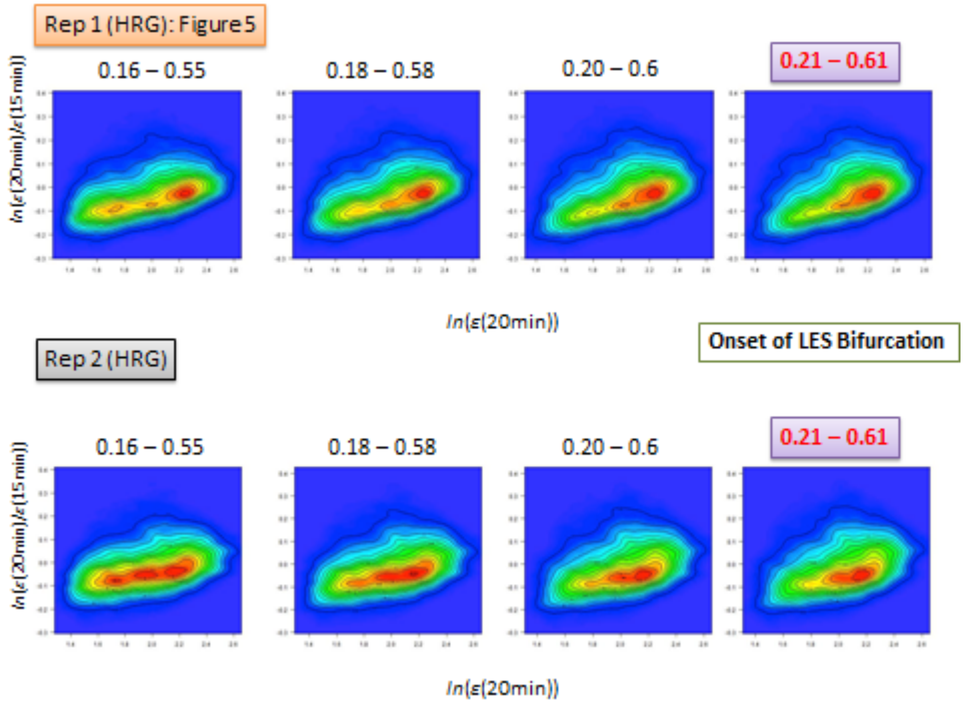
Rep 1 (HRG): Figure 5
0.16 – 0.55
0.18 – 0.58
0.20 – 0.6
0.21 – 0.61
ln(ε(20min)/ε(15 min))
ln(ε(20min))
Onset of LES Bifurcation
Rep 2 (HRG)
0.16 – 0.55
0.18 – 0.58
0.20 – 0.6
0.21 – 0.61
ln(ε(20min)/ε(15 min))
ln(ε(20min))

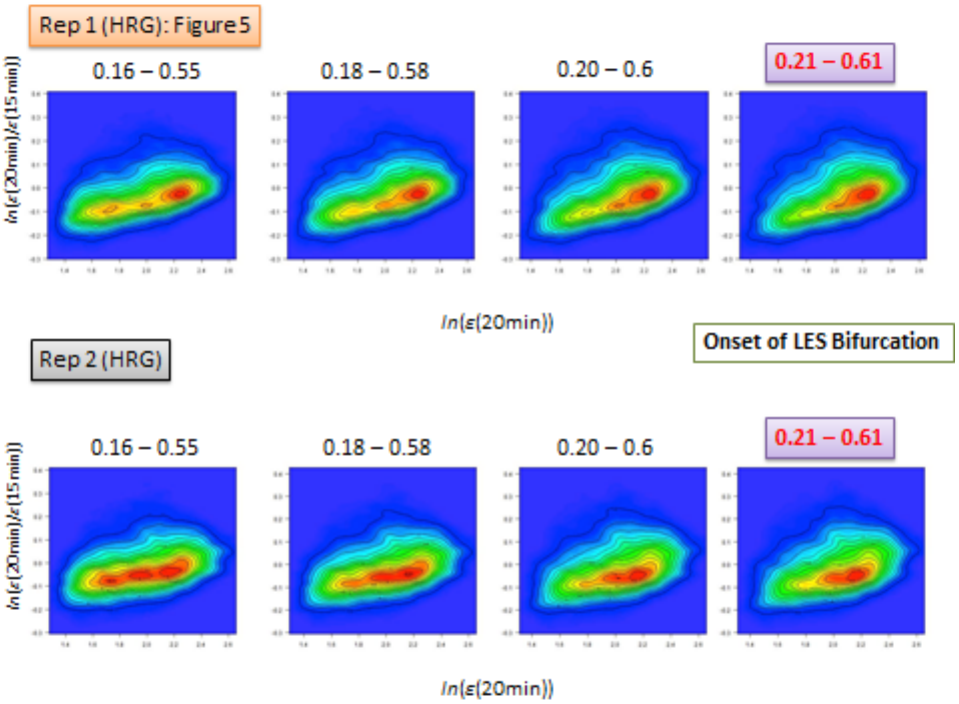

Rep 1 (HRG): Figure 5
0.16 – 0.55
0.18 – 0.58
0.20 – 0.6
0.21 – 0.61
ln(ε(20min)/ε(15min))
ln(ε(20min))
Onset of LES Bifurcation
Rep 2 (HRG)
0.16 – 0.55
0.18 – 0.58
0.20 – 0.6
0.21 – 0.61
ln(ε(20min)/ε(15min))
ln(ε(20min))

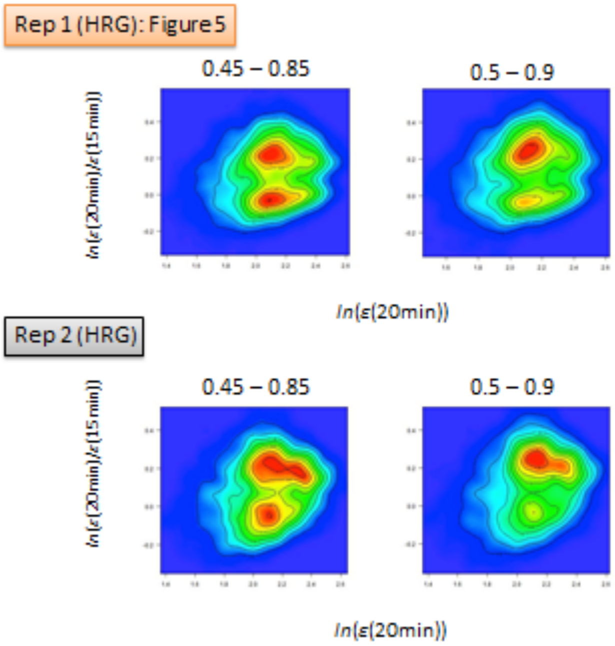

Rep 1 (HRG): Figure 5
0.45 – 0.85
0.5 – 0.9
ln(ε(20min)/ε(15min))
ln(ε(20min))
Rep 2 (HRG)
0.45 – 0.85
0.5 – 0.9
ln(ε(20min)/ε(15min))
ln(ε(20min))

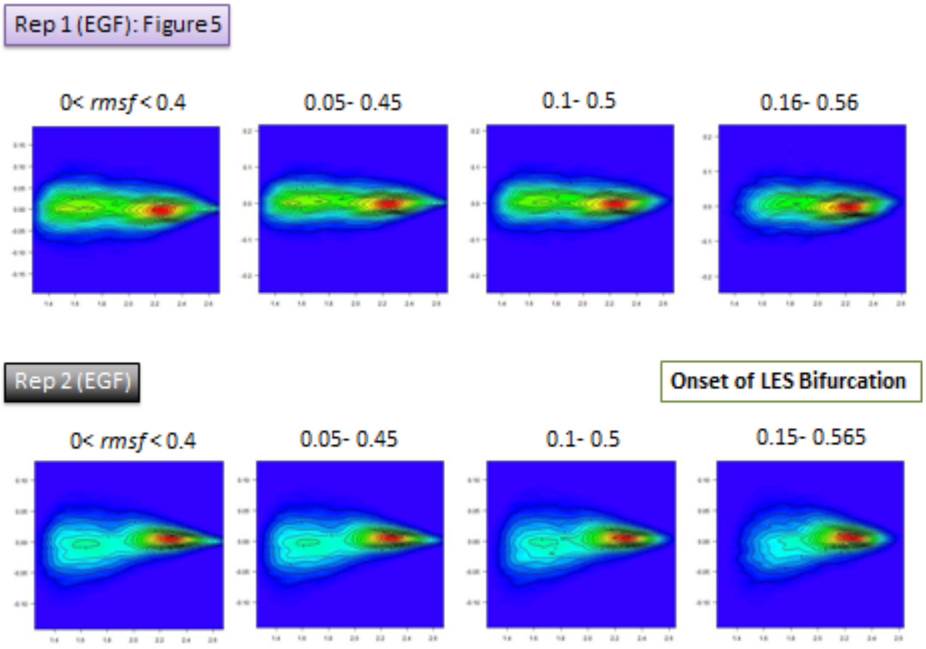
Rep 1 (EGF): Figure 5
0< *rmsf* < 0.4
0.05- 0.45
0.1- 0.5
0.16- 0.56
Rep 2 (EGF)
Onset of LES Bifurcation
0< *rmsf* < 0.4
0.05- 0.45
0.1- 0.5
0.15- 0.565

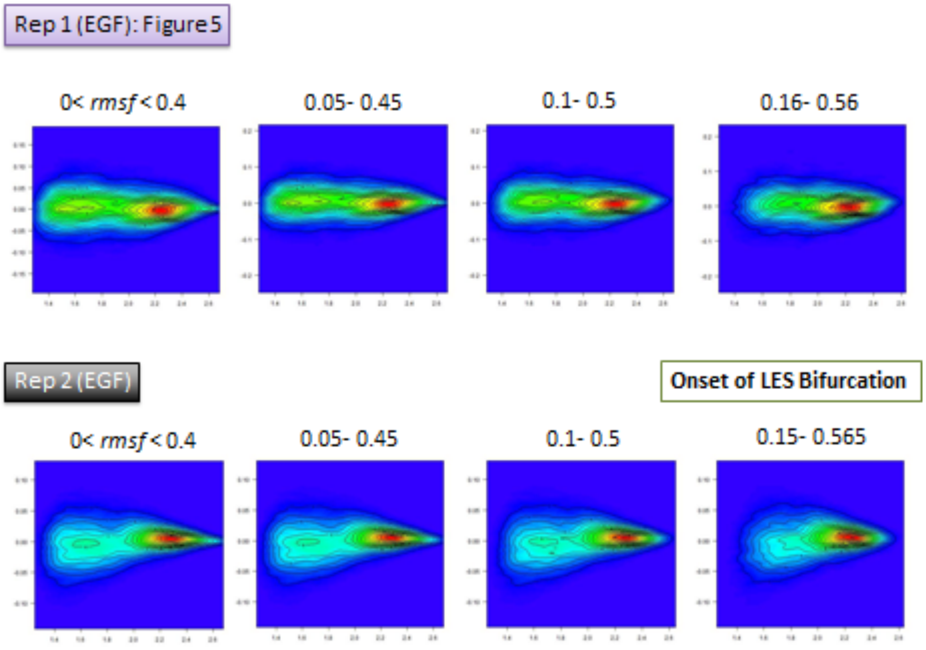
Rep 1 (EGF): Figure 5
0< *rmsf* < 0.4
0.05- 0.45
0.1- 0.5
0.16- 0.56
Rep 2 (EGF)
Onset of LES Bifurcation
0< *rmsf* < 0.4
0.05- 0.45
0.1- 0.5
0.15- 0.565
